# NMR Molecular Replacement Provides New Insights into Binding Modes to Bromodomains of BRD4 and TRIM24

**DOI:** 10.1021/acs.jmedchem.1c01703

**Published:** 2022-03-31

**Authors:** Felix Torres, Reto Walser, Janina Kaderli, Emanuele Rossi, Romel Bobby, Martin J. Packer, Sunil Sarda, Graeme Walker, James R. Hitchin, Alexander G. Milbradt, Julien Orts

**Affiliations:** † Swiss Federal Institute of Technology, Laboratory of Physical Chemistry, HCI F217, Eidgenossische Technische Hochschule Zurich, Vladimir-Prelog-Weg 2, 8093 Zürich, Switzerland; ‡ BioPharmaceuticals R&D, AstraZeneca, Cambridge, CB4 0WG, United Kingdom; # Oncology R&D, AstraZeneca, Cambridge, CB4 0WG, United Kingdom; ○ Drug Discovery Unit, Cancer Research UK Manchester Institute, Alderley Park, Macclesfield SK10 4TG, United Kingdom; □ Department of Pharmaceutical Sciences, University of Vienna, Althanstrasse 14, A-1090 Vienna, Austria

## Abstract

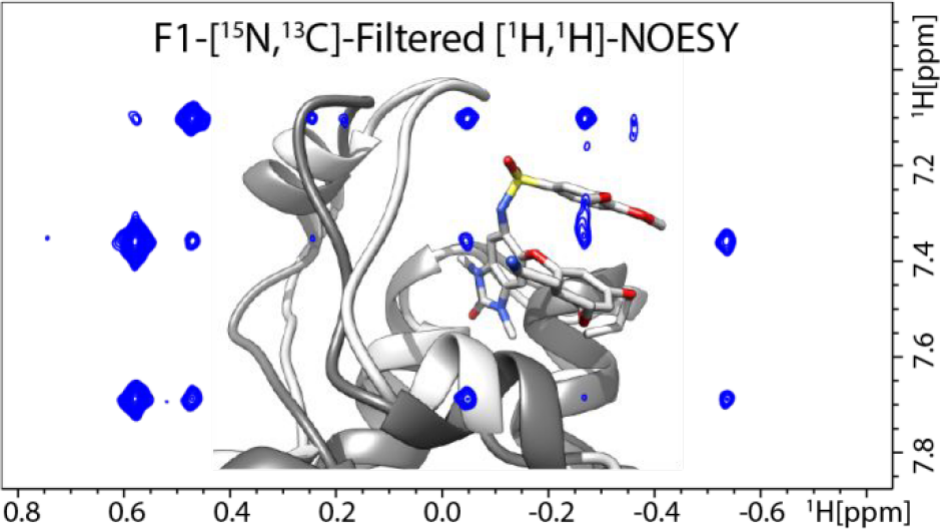

Structure-based drug discovery (SBDD) largely relies on structural information from X-ray crystallography because traditional NMR structure calculation methods are too time consuming to be aligned with typical drug discovery timelines. The recently developed NMR molecular replacement (*N*MR^2^) method dramatically reduces the time needed to generate ligand–protein complex structures using published structures (apo or holo) of the target protein and treating all observed NOEs as ambiguous restraints, bypassing the laborious process of obtaining sequence-specific resonance assignments for the protein target. We apply this method to two therapeutic targets, the bromodomain of TRIM24 and the second bromodomain of BRD4. We show that the *N*MR^2^ methodology can guide SBDD by rationalizing the observed SAR. We also demonstrate that new types of restraints and selective methyl labeling have the potential to dramatically reduce “time to structure” and extend the method to targets beyond the reach of traditional NMR structure elucidation.

## Introduction

Bromodomains (BDs) specifically recognize the acetylation state of lysine side chains (Kac) within histone proteins, an important posttranslational modification.^1−5^ The human genome encodes 46 proteins, spread over eight families, that contain more than 60 different BDs involved in epigenetic regulation but also in various diseases such as inflammation, diabetes, neurological diseases, and cancer through the deregulation of transcription factors.^6−15^ These findings make epigenetic targets and bromodomains, in particular, a relevant field of research for cancer therapeutics.^7,11−13,16,17^ Previous work already reported the bromodomain and extraterminal (BET) proteins being directly involved in cancer and validated BET proteins as targets for chemotherapies.^7−9,15,18−20^

Transcription intermediary factor 1 proteins (TIF1) are a subgroup of proteins from the tripartite motif (TRIM) family, also known as the RBCC family.^21,22^ The canonical TRIM is composed of three zinc-binding domains [a RING (R), a B-box type 1 (B1), and a B-Box type 2 (B2)] followed by a coiled-coil (CC). C terminal to the TRIM motif additional domains may be found, such as the PHD-BD motif (PHD, plant homeodomain) found in TRIM24 (also known as TIF1alpha).^23,24^ Through the activity of its N-terminal RING domain, TRIM24 acts as an E3 ubiquitin ligase, negatively regulating p53 stability.^25^ Deregulation of E3 ubiquitin ligases is commonly observed in human cancers.^26^ Furthermore, a link between TRIM24 and breast cancer has been established,^27^ and in the same study, TRIM24 was identified as a reader of the dual-histone mark H3-K4me0K23ac, i.e., a histone 3 N-terminal tail which is unmodified at K4 and acetylated at K23, suggesting its role as an epigenetic “reader” protein.

The bromodomain containing protein 4 (BRD4) has two bromodomain reader modules that specifically recognize the acetylation state of histone lysine side chains. The discovery of iBET-762 and other classes of small molecules demonstrated that BRD4 can be targeted by blocking the acetyl lysine binding pockets of its bromodomains.^28^ Structure-based drug discovery has been extensively used to guide the design of these bromodomain inhibitors with over 300 BRD4 BD1–compound complexes reported in the literature. However, there is a paucity of structural information for BRD4 BD2 as highlighted by only a few X-ray-derived entries in the public domain, suggesting BD2 might be less amenable to X-ray diffraction. Therefore, the second bromodomain of BRD2 has often been used as a structural surrogate as it has a higher homology to BRD4 BD2 than BRD4 BD1 has to BRD4 BD2.

X-ray crystallography is the most widely used technique for structure-based drug design (SBDD).^29,30^ In the absence of an X-ray structure, NMR can provide an alternative, but its low throughput is a major drawback and usually does not match the expected timelines of medicinal chemistry projects. We recently developed the *N*MR^2^ technique, a molecular replacement-like approach using NMR to rapidly determine ligand–protein complex structures at the binding site with atomic resolution.^31−34^ Clear advantages of the method are that it by-passes the long and tedious protein resonance assignment step and harnesses synergies with other structure determination techniques such as X-ray crystallography. The *N*MR^2^ technique calculates complex structures in a fully automated way using unassigned sparse NOE data and a structural model of the apo state of the receptor, in the above cases derived from X-ray crystallography. Here, we report the structure of the bromodomains of TRIM24 and BRD4 BD2 in complex with small molecule ligands and a new protocol of *N*MR^2^ structure determination using specifically labeled methyl groups and anti-NOEs. We show that the *N*MR^2^ method is able to both rationalize SAR data as it is commonly generated in a SBDD project and deliver structures within the timeframes encountered in a typical hypothesis–synthesis–testing cycle of a medicinal chemistry project.

## Results and Discussion

**Figure 1:**
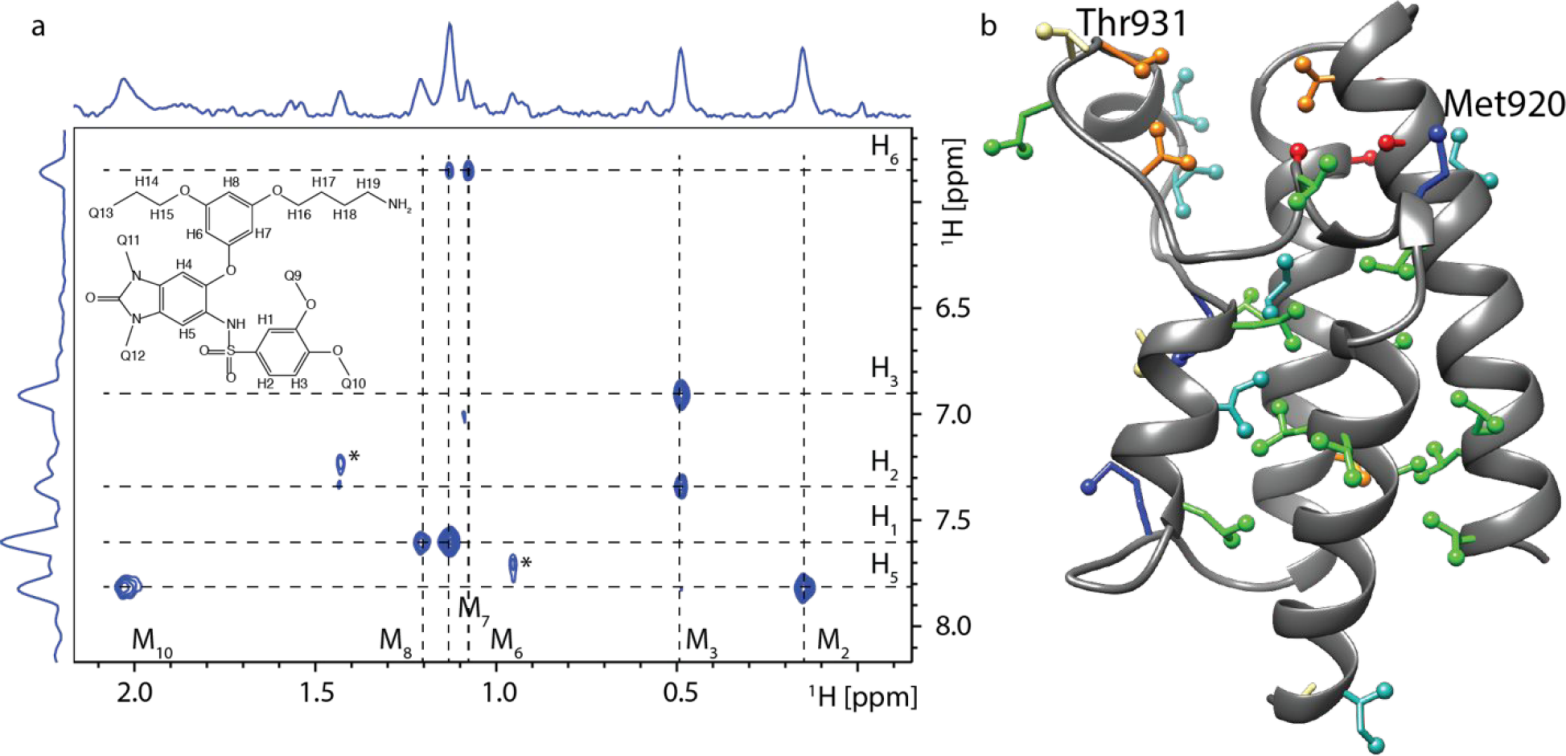
(a) Methyl–aromatic region of the [^15^N,^13^C]-filtered [^1^H,^1^H]-NOESY spectrum of TRIM24 (1.1 mM) with the ligand (1:1) measured on an 800 MHz spectrometer at a 100 ms mixing time. Proton names for the ligand are reported in the inset 2D representation of the ligand structure, and methyl groups of the protein are arbitrarily called M#. (b) Ribbon representation of the bromodomain of TRIM24 used as the starting protein structure for the *N*MR^2^ complex structure calculations (PDB code 4YC9). Amino acid residues containing methyl groups are depicted with sticks, and methyl groups are depicted with spheres (Ala, red; Ile, cyan; Leu, green; Met, blue; Thr, yellow; Val, orange). Met920 and Thr931 are labeled for clarity.

For compound **1** the ligand core structure composed of the dimethoxyphenyl group is structurally well characterized with the two aromatic rings stacked and exhibiting numerous NOEs to the protein (Figure 1a). On the other hand, the aliphatic tail ending with an ammonium group does not exhibit any NOEs, suggesting that this part of the compound is solvent exposed. Because only one methionine and one threonine are present in the binding site (Figure 1b), Met920 and Thr931 can be easily assigned.

**Figure 2:**
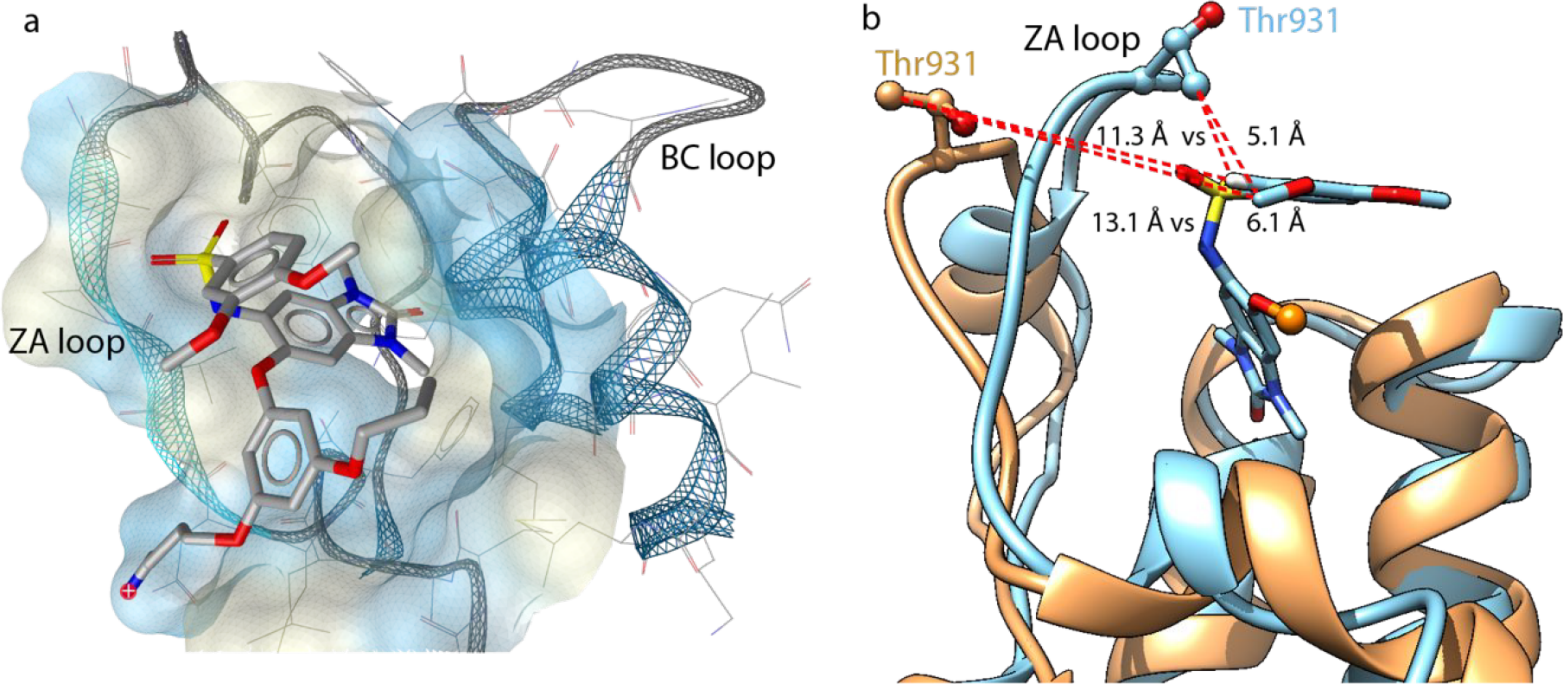
(a) Ribbon representation of TRIM24 in complex with compound **1**. Protein ribbons are depicted in blue and gray. Side chains are represented as lines, and ligand is represented as sticks. Protein surface is colored according to the aggregated atoms hydrophobicity with yellow being more hydrophobic compared to blue patches that are hydrophilic. (b) Comparison between the *N*MR^2^ structure (blue) and the X-ray structure (orange) of TRIM24 in complex with compound **1** (PDB code 7B9X) and a close analog of compound **1**, IACS-9571 (PDB code 4YC9, Supplementary Figure S1). Compound **1** is depicted as blue sticks. Aromatic moiety containing the aliphatic chain ending with the ammonium group is not shown for clarity and is replaced here by an orange sphere. Two observed intermolecular NOE interactions between the methyl group of T931 and the ligand are marked with red dashed lines, and corresponding distances measured for the two structures are reported side by side.

The interactions between compound **1** and TRIM24 are in line with the already reported 3D structure derived by X-ray crystallography,^35^ namely, the dimethylbenzimidazolone is deeply buried in the center of the binding site, and the two dimethoxyphenyl groups are stacking above the helix from the ZA loop and flanked by the ZA loop and the N-terminal α-helix 1 (Figure 2a). During the NMR structure calculation protocol, the ZA loop had to be flexible to prevent strong protein–ligand intermolecular NOE violations. This can be seen on the superposition of the initial X-ray structure and *N*MR^2^ structure of the complex, where the distances between the Thr931 and the ligand protons H1 and Q9 are reduced by approximately one-half in the *N*MR^2^ complex structure (Figure 2b). The *N*MR^2^ structure calculation protocol first assumes the backbone of the protein being unperturbed upon binding of the ligand, while the side chains are fully flexible over the whole calculation protocol. However, in the case of TRIM24 with compound **1**, two distance restraints were severely violated. While the *N*MR^2^ protocol still converged to the correct structure, we refined the complex structure by allowing protein flexibility and refined the structure in explicit water solvent with full electrostatic potential. The NOE distance restraints were implemented as collective variables, and the protein backbone was slightly restrained to its initial position, taken from the PDB structure 4YC9, by harmonic restraint potentials. The ZA loop could then bend toward the ligand to fulfill the experimentally derived distance restraints (Figure 2b). The ZA loop has previously been shown to be highly flexible as inferred from crystallographic studies,^36,37^ NMR relaxation experiments,^38^ and molecular dynamics simulations.^39,40^ Our findings further support that the ZA loop undergoes large conformational changes and provides structural insight into the closed loop state of the ZA loop in the ligand bound form of the TRIM24 bromodomain.

The *N*MR^2^-derived complex structure of compound **1** bound to the TRIM24 bromodomain maintains the two core interactions identified in the X-ray structure between the *N*-dimethylated analogue of compound 1, IACS-9571, and the TRIM24 bromodomain: The ionic interaction of the charged amine with Asp926 and the canonical interaction between the side chain of Asn980 and the benzimidazolone core of compound **1** or IACS-9571.^35^ In addition, the *N*MR^2^ structure is consistent with the described SAR observed for the sulfonamide substituent. X-ray structures show no interaction between the sulfonamide moiety of the compound and the protein. Yet, the SAR reported by Palmer et al. showed exquisite sensitivity to changes to the sulfonamide, and its removal resulted in a >20-fold loss in potency.^35^ The *N*MR^2^ structure shows a clear interaction between Thr931 and the sulfonamide group, explaining the observed SAR for this group. It is interesting to note that the ZA loop folds over the ligand in a lid-like fashion. This is a previously unobserved feature for bromodomains, resulting in a more closed binding pocket. TRIM24 was classified as challenging in terms of “druggability” in a study comparing the structures of all known bromodomains.^41^ The observed high affinity of compound **1** and its derivatives toward the TRIM24 BD was rather surprising in that light. The observation of a more closed structure through the closure of the ZA loop over the ligand binding pocket reconciles this surprising finding. Indeed, the druggability of the closed TRIM24 binding pocket increases significantly according to the algorithm Sitemap (Supplementary Table S1).^42^

**Figure 3:**
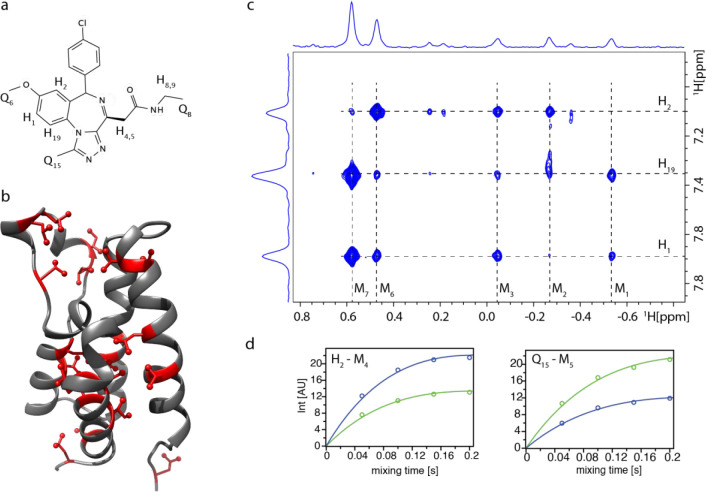
(a) Two-dimensional representation of the ligand structure of iBET-762 with the corresponding proton names involved in NOEs. (b) Ribbon representation of BRD4 BD2 used as the starting protein structure for the *N*MR^2^ complex structure calculations (PDB code 6U8I). Amino acid residues Ile, Leu, and Val are depicted with red sticks, and methyl groups are depicted with spheres. (c) Methyl–aromatic region of the [^15^N,^13^C]-filtered [^1^H,^1^H]-NOESY spectrum of BRD4 BD2 (350 μM) with the ligand (1:1) measured on an 800 MHz spectrometer at a 200 ms mixing time. (d) NOE build ups for the intermolecular NOE cross-peaks measured at mixing times 50, 100, 150, and 200 ms and from both sides of the NOESY spectra diagonal (blue and green curves). Build-up curves were fitted using eq 1. Proton assignments for the ligand are reported in a, and methyl groups of the protein are arbitrarily called M#.

We then further explored the capabilities of the *N*MR^2^ method to provide structural information with a turnaround time suitable for medicinal chemistry projects using BRD4 BD2 in complex with iBET-762 (Figure 3a and 3b). For this system, the *N*MR^2^ protocol relied solely on accurate distances derived from NOEs. As such it did not include the acquisition of any other experimental data other than the essential set of NOESY spectra (Figure 3c), reducing the time for spectra acquisition, processing, and analysis considerably. Several methods have been developed to control or correct for spin diffusion, as it compromises the accuracy of NOE-derived distance restraints.^43,44^ A straightforward approach is to remove surrounding protons by deuteration. Specific labeling schemes with reprotonated methyl groups in a deuterated background have been extensively used to study large molecular complexes.^45,46^ Methyl groups are particularly interesting because of their abundance, sharp resonance lines, and even spread throughout the protein structure as is evident also for BRD4 BD2 (Figure 3b). Here we exploit these properties for SBDD by specifically ^13^C,^1^H labeling the BRD4 BD2 domain on the methyl groups of the isoleucine (δ1 only), leucine, and valine amino acid residues in an otherwise fully ^12^C,^2^H background. Generating proteins with such labeling pattern has become straightforward in *E. coli*-based expression systems through the use of commercially available precursors.^47^ In addition, this labeling scheme reduces spin diffusion, provides excellent NOE build-up curves (Figure 3d), and leads to NOESY peak patterns showing high signal-to-noise ratios.

Although the traditional approach to derive structures by NMR spectroscopy does not consider missing NOE cross-peaks as usable information, they nonetheless can contain structural information. Overall, we could add another 37 anti-NOE restraints, raising to a total of 76 protein–ligand restraints, improving the convergence and speed of the *N*MR^2^ calculations. Furthermore, since the total amount of structural information increased, we could also increase the tolerance on the calibration of the NOE restraints as shown from the distance calibrations on the two bromodomain systems in Supplementary Tables S2 and S3. This improves the robustness of the *N*MR^2^ method by reducing errors that could stem, for example, from nonoptimal NMR experiment parameters, bad cross-peak integration, or wrong estimation of the complex correlation time.

Finally, the half-filtered NOESY experiment provides the possibility to group the prochiral methyl groups of the Leu and Val amino acid residues based on the strong intraresidue methyl–methyl NOE observed in the protein–protein NOESY spectrum.

**Figure 4:**
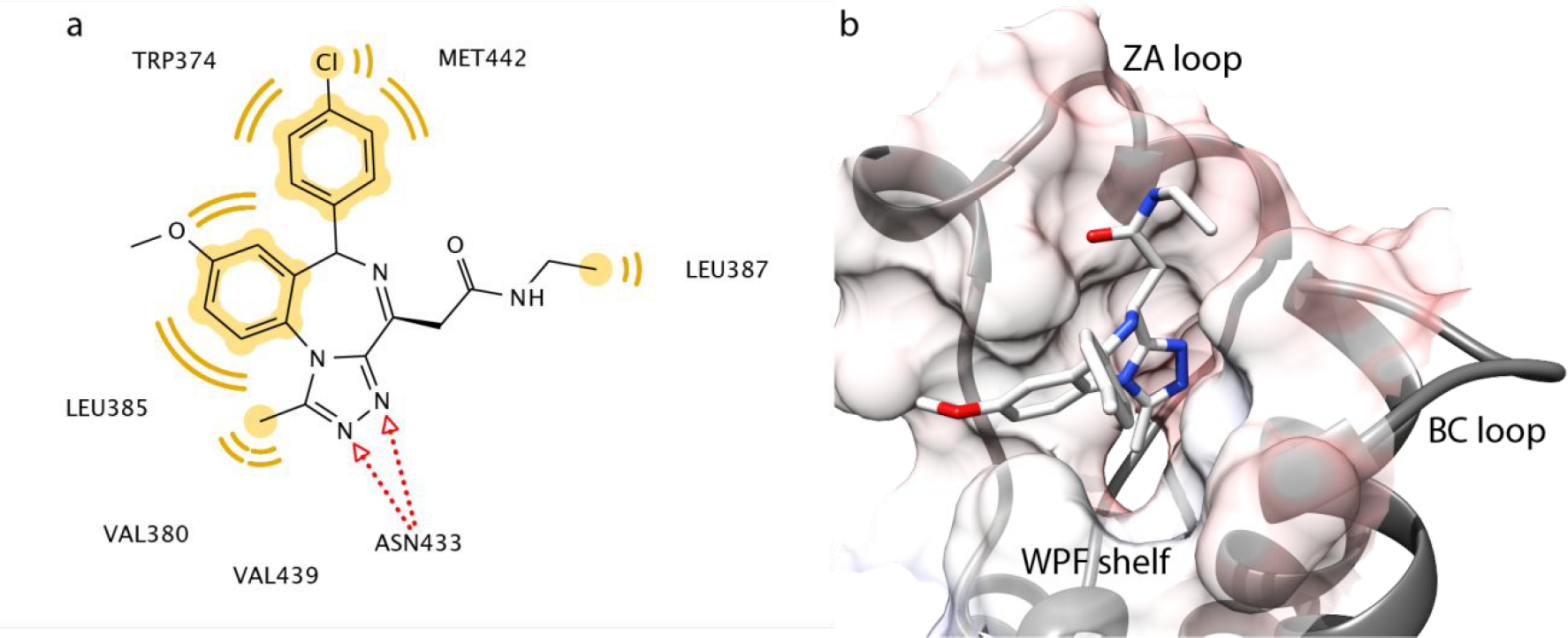
(a) Stick representation of iBET-762 and its interactions with BRD4 BD2. Yellow coloring represents the hydrophobic interactions between the ligand and the protein. Red arrows depict intermolecular H bonds. (b) Ribbon representation of the *N*MR^2^ structure of BRD4 BD2 in complex with iBET-762 (PDB code 7AQT). Protein ribbons are depicted in gray, ligand is represented with sticks, and protein surface is shown as semitransparent solid and shaded according to the electrostatic potential.

The presented *N*MR^2^ structure of BRD4 BD2 in complex with iBET-762 nicely recapitulates the conserved interactions that have been reported for this class of compounds sharing the same scaffold. The methyl group of iBET-762 engages with the small hydrophobic methyl-recognition pocket, centered around Phe376 and flanked by Val439, while the nitrogens of the triazole moiety are located within hydrogen-bond distance to the side chain of conserved Asn433 (Figure 4, Supplementary Figure S2).^18^ In addition, the fused phenyl moiety extends into the ZA channel with contacts to Leu385 on one side and to Pro375 on the other side of the channel, while the pendent chlorophenyl is engaging Trp374 and Pro375 of the WPF shelf, as previously observed.^18^

With a short computational time and only a few days for sample preparation and data acquisition, the presented NMR method is well within the time frame to support typical design–make–test–analyze (DMTA) cycles.

## Conclusions

We demonstrated that the NMR Molecular Replacement methodology can generate ligand–protein complex structures useful for SBDD. A high-affinity ligand for the TRIM24 bromodomain, IACS-9571, was recently published by a research group from the MD Anderson Cancer Center.^35^ This benzimidazolone compound displays a remarkably high affinity (*K*_D_ = 1.3 nM). This ligand represented a breakthrough in lead generation against bromodomains with low predicted druggability scores, such as the TRIM24 BD.^41^ The *N*MR^2^ structure of TRIM24 in complex with compound **1**, a close analogue of IACS-9571, recapitulates the key interactions observed in the X-ray structures of TRIM24 BD and IACS-9571. At the same time the *N*MR^2^ structure shows that the dynamic features of the ZA loop play an important role in binding ligands with high affinity and that these features are missed in the X-ray crystallographic structure. In solution, the ZA loop folds across the ligand’s sulfonamide core and shows close-range interactions between T931 and the sulfonamide group of the compound. This sulfonamide was found to be exquisitely sensitive to changes in substitution,^35^ a puzzling bit of SAR when looking at the X-ray structure of bound IACS-9571, which however makes perfect sense in light of the NMR structure we present here. We speculate that the movement of the ZA loop and the concomitant formation of the more closed pocket directly translates into a higher druggability score (Supplementary Table S1).

Interestingly, the reported selectivity profile of the benzimidazolone series can be rationalized also by our reported structure. Closure of the ZA loop over the ligand binding pocket occurs through a hinging motion at the N and C termini of the ZA loop around residues 930 and 942, respectively. This is evident from significant differences in backbone dihedral angles between the X-ray and NMR structures in these regions (Supplementary Figure S3a). Whereas the C terminus of the ZA loop is very conserved among bromodomains, significant differences exist at the N terminus. Interestingly the two bromodomains having the highest affinity of IACS-9571 both have a unique Pro-Leu motif at this position (Supplementary Figure S3b). All other bromodomains showing an appreciable affinity toward IACS-9571 carry a Ser/Asn-Leu motif. Taken together, the Pro/Ser/Asn-Leu motif at the N terminus of the ZA loop is a unique feature among the 32 tested bromodomains showing affinity for IACS-9571. We propose this motif to be responsible for high affinity against compounds of the type of IACS-9571.

The presence of two unique methyl-containing amino acids (Met920 and Thr931) in the binding pocket of TRIM24 BD allowed us to treat some of the measured NOEs as classical, nonambiguous restraints. This allowed for increased speed and convergence of the *N*MR^2^ algorithm. In the absence of such unambiguously assigned anchor residues in the binding pocket, a TOCSY-based classification of amino acids into different families (Thr, Ala, or Ile/Leu/Val) can also serve a similar purpose, albeit at the expense of a longer NMR acquisition time. Such experimental time (commonly on a high-field magnet), time required for processing, and distance restraint extraction and computational time for the *N*MR^2^ algorithm make up the total time required to derive a structure. This total “time to structure” is ultimately the figure of merit governing whether a method is applicable to the classical DMTA cycles in SBDD. The exact time required will always depend to a large extent on the protein target’s size and complexity, but for traditional SBDD targets, obtaining a protein–ligand structure within ca. 1 week is well possible.

We thus sought to explore further ways to lower this “time to structure” and to explore the more general utility of the *N*MR^2^ method. We tested the applicability of the *N*MR^2^ method to selectively methyl-protonated systems in an otherwise highly deuterated background. Such labeling schemes can greatly extend the accessible molecular weight range for NMR experiments.^45,48^ As such, it presents in our view the most general method to make the *N*MR^2^ method applicable to targets of all sizes. Our *N*MR^2^-derived structure is the first report of an iBET-762 BRD4 BD2 complex. Hitherto, only structures of iBET-762 in complex with BRD4 BD1 (PDB code 3P5O), BRD2 BD1 (PDB code 2YEK), and BRD2 BD2 (PDB code 5DFC) have been published. Although iBET-762 has been shown to bind to various members of the BET family, published data suggests that the interaction of iBET-762 with BRD4 BD2 has one of the highest affinities among members of the BET family,^18,49^ underlining the relevance of this interaction.

We introduced anti-NOEs for the *N*MR^2^ method to gather more structural restraints driving the structure calculations. The absence of an NOE between two nuclei is not strictly speaking proof that the two nuclei are not close to each other in space. However, in the case where two nuclei are indeed far from each other in space, this information contains valuable information that could be harnessed in structure calculation. This has been realized by other groups and has found different application, e.g., in automated resonance assignment generation.^50,51^ We sought to make use of such anti-NOE restraints in a very conservative fashion. We only accepted the use of an anti-NOE where both individual resonances are clearly visible and both individual resonances show NOEs to other resonances. Anti-NOEs contain a wealth of information and have the potential to significantly improve both the convergence and the accuracy of structure calculation, especially when the restraints are ambiguous like in *N*MR^2^. While the information content of an anti-NOE is lower compared to that of a visible NOE, the number of anti-NOEs is by far more important. Finally, we anticipate that the use of anti-NOEs will reach its full potential in cases when the ligand can freely sample a large conformational space within the binding site or when the ligand has a low molecular weight (e.g., fragments).

In summary, we reported the structures of two bromodomains complexed with inhibitors using the *N*MR^2^ methodology. We were able to show that the method can explain SAR as commonly generated in SBDD campaigns. We further improved the speed and robustness of the method, leading to as little computation time as a few minutes on a single desktop machine. Together with a high signal-to-noise due to selective methyl labeling, this makes it well suited for the short DMTA cycles encountered in drug discovery. The bottleneck for NMR structure-based drug design is not any longer the analysis of the NMR data but the time required for sample preparation and the data acquisition time. In this study, the complete *N*MR^2^ workflow (including sample preparation) took less than 2 weeks. We envision that *N*MR^2^ will become an important tool in SBDD that can be used by a broad population of users that are not NMR specialists. *N*MR^2^ has the potential to become a new standard in structure-based drug design, complementing the current gold standard, X-ray crystallography, or the newly emerging field of cryo-electron microscopy. Finally, the approach could inspire future development of fully automated de novo NMR structure determination.

## Experimental Section

### Synthetic Chemistry

Compound **1** was synthesized following a reported protocol,^35^ and the discovery of iBET-762 has been described in detail.^28^ Both compounds are >95% pure by HPLC analysis (Supplemental Figure S4 for iBET-762 and Supplemental Figure S5 for compound **1**).

### Protein Expression and Purification

A uniformly ^13^C,^15^N-labeled construct comprising the TRIM24 bromodomain was expressed and purified as described previously.^52^

The second bromodomain of human BRD4 (Uniprot ID O60885; residues H341–E460) was cloned into a pET28 vector (Novagen). The protein was expressed in *E. coli* BL21 Gold (DE3) cells in a D_2_O-based M9 medium containing 1 g/L ^15^NH_4_Cl and 2 g/L ^12^C-glucose-*d*_7_. For selective methyl protonation at Ile-δ1, Leu-δ1/2, and Val-γ1/2, the growth medium was supplemented with 70 mg/L 2-ketobutyric acid-4-^13^C sodium salt hydrate (Isotec) and 120 mg/L 2-keto-3-(methyl-^13^C)-butyric-4-^13^C acid sodium salt (Isotec) 30 min prior to induction. The expression medium contained 50 μg/mL kanamycin, and overexpression of protein was induced by the addition of 1 mM IPTG at an OD_600_ of 0.6. Cultures were grown overnight at 18 °C before harvesting. Cells were resuspended in 50 mM TRIS, pH 8.0, 300 mM NaCl, 1 mM β-mercaptoethanol, 10 mM imidazole, Complete Protease Inhibitor tablets (Roche), and benzonase nuclease (2.5 U/mL). Resuspended cells were lysed using a Constant Systems cell disruptor at 25 000 psi and clarified by centrifugation at 35 000*g* for 60 min at 4 °C. BRD4 BD2 was purified from the supernatant by nickel-affinity chromatography followed by treatment with TEV protease. Protein was further purified by size-exclusion chromatography using a Superdex 75 10/300 GL column in 20 mM HEPES, pH 7.4, 100 mM NaCl, 1 mM TCEP and subsequently concentrated to the required concentrations using centrifugal concentrators.

The identity of both proteins used in this work was confirmed by ESI-MS, and purity was >95% as judged by SDS-PAGE.

### NMR Sample Preparation

The NMR sample for TRIM24 contained 1.1 mM uniformly ^15^N- and ^13^C-labeled protein in 95% H_2_O/5% D_2_O at pH 7.4 in 50 mM HEPES, 100 mM NaCl, 1 mM TCEP. Compound **1** was added in equimolar amounts from a 20 mM stock in DMSO-*d*_6_. Resonance assignments of compound **1** were obtained on a 1 mM sample in the same buffer as above.

The NMR sample for BRD4 BD2 contained 350 μM ILV and otherwise uniformly ^12^C,^2^H-labeled protein in 100% D_2_O at pH 6.7 in 50 mM Na_2_HPO_4_, 0.5 mM d-TCEP. iBET-762 was added in 1.2 molar excess with respect to the protein. Excess unbound compound was subsequently removed by passing the sample over a PD-10 desalting column.

### NMR Measurements

All NMR experiments involving protein samples were recorded at 303 and 298 K for TRIM24 and BRD4 BD2, respectively, on an 800 MHz Bruker Avance III spectrometer equipped with a 5 mm TCI cryoprobe with *z*-axis gradients running Topspin 3.2 in 3 mm NMR tubes. NMR experiments for resonance assignment of compound **1** and iBET-762 were carried out at 303 K on a 600 MHz Bruker Avance III spectrometer equipped with a 5 mm TCI cryoprobe with *z*-axis gradients running Topspin 3.5 in 5 mm NMR tubes.

For TRIM24 bound to compound **1**, a series of nine double-half-filtered NOESY experiments was measured with mixing times of 10, 20, 30, 40, 50, 60, 80, 90, 100, and 120 ms.^53^ Each experiment was acquired with 2048 × 512 complex points in the direct and indirect dimensions, respectively, for an experimental time of ca. 7 h per spectrum. The spectra covered 9.5 kHz in both dimensions. Resonances of compound **1** were assigned with the help of DQF-COSY, ROESY (250 ms), and TOCSY (80 ms) spectra. All homonuclear 2D compound spectra were acquired with 2048 × 512 complex points, 32 scans per increment, and covering 6 kHz spectral widths in both dimensions.

A 2D constant-time ^13^C HSQC experiment was acquired with 1024 × 120 complex points in the direct and indirect dimensions, respectively, 4 scans per increment, and a 13.3 ms constant-time delay. The spectrum covered 10.5 and 13.6 kHz in the proton and carbon dimensions, respectively.

A 3D HCCH-TOCSY experiment was recorded with 2048 × 60 × 66 complex points in the direct and the two indirect dimensions, respectively, and with 8 scans per increment, covering 11.2, 15.1, and 11.2 kHz in each of the dimensions. A 22 ms DIPSI-3 sequence was used for isotropic mixing in the ^13^C dimension.

For BRD4 BD2 bound to iBET-762, a series of 4 double half-filtered NOESY experiments employing an IPAP element to acquire all four combinations of ^12^C /^13^C filtering with mixing times of 50, 100, 150, and 200 ms was recorded. Each experiment was acquired with 2048 × 512 complex points in the direct and indirect dimensions, respectively, for an experimental time of ca. 35 h per spectrum. The spectra were recorded with 16 scans per increment per double-half-filter experiment and covered 11 and 9.5 kHz in the direct and indirect dimensions, respectively. Resonances of iBET-762 were assigned with the help of double-half-filtered DQF-COSY acquired with 2048 × 300 complex points, 16 scans per increment, and covering 12.5 and 9.5 kHz in the direct and indirect dimensions, respectively. Double-half-filtered NOESYs were also used for compound resonance assignment.

### *N*MR^2^ Structure Determination

All spectra were processed with Topspin 3.1 (Bruker) and evaluated with ccpNMR analysis 2.4.^54^ Distances were derived from NOE build-up curves using a simple two-spin system model (*i*,*j*) and following the established protocol.^31,46,55,56^ The autorelaxation rates, *ρ*_*i*_, and initial magnetizations, *ΔM*_*ii*_(0), were determined using a monoexponential decay function, *ΔM*_*ii*_(*t*) = *ΔM*_*ii*_(0)exp(−*ρ*_*i*_*t*). The cross-relaxation rates, *σ*_*ij*_, were fitted following a two-spin system approximation model for the protein–ligand NOEs, *ΔM*_*ij*_(*t*), eq 1. The corresponding distances, *r*_*ij*_, were derived from the cross-relaxation rates, *σ*_*ij*_, defined in eq 3
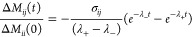
1
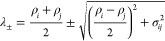
2
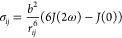
3
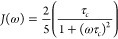
4
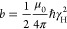
5where *μ*_*0*_ is the permeability of vacuum, *ℏ* the reduced Planck constant, γ_H_ the gyromagnetic ratio of the nucleus, and τ_*c*_ the rotational correlation time of the protein. Rotational correlation times of 10.5 ns for TRIM24 and 10.1 ns for BRD4 BD2 were derived from ^15^N-*T*_1_ and ^15^N-*T*_1ρ_ relaxation rates using the software TENSOR2.^57^

#### TRIM24

Interaction between the TRIM24 BD and compound **1** was in the slow exchange regime on the NMR chemical shift time scale, indicating a submicromolar affinity, analogous to the behavior observed for the closely related compound IACS-9571 for which a nanomolar affinity was reported (Supplementary Figure S6).^35^ For the compound **1**—TRIM24 complex, we could derive a dense network of 19 intraligand distances from the intraligand NOE cross-relaxation rates, free of spin diffusion based on the build-up curves. Twenty-four protein–ligand intermolecular cross-relaxation rates, also free of spin diffusion, could be determined and subsequently converted to distance restraints (Supplementary Table S2). Using ^13^C constant-time HSQC and 3D ^13^C-resolved TOCSY spectra, partial methyl assignments were readily derived, such as for the Thr931 and the Met920 methyls, due to their characteristic chemical shift relative to the more ubiquitous Ile, Val, and Leu methyl groups.^58^ The methionine methyl resonances can be recognized using a constant-time ^13^C-HSQC spectrum because methionine methyl groups do not evolve under any scalar carbon–carbon coupling and have an opposite sign to other methyl groups (Supplementary Figure S7). The intermolecular NOEs between compound **1** and the methionine as well as the threonine methyl resonances were not treated as semiambiguous but like a classically assigned NOE. Having such an anchor point in the binding site helps the *N*MR^2^ algorithm considerably both by improving convergence and by reducing computational time. The methyl resonance assignment of the only threonine present in the binding site, Thr931, was easily derived using the 3D ^13^C-resolved TOCSY. Furthermore, some of the methyl peaks can be identified as stemming from alanine residues as opposed to leucine, valine, and isoleucine amino acid residues.^58^ This amino acid type classification further increases the speed and convergence of the computational algorithm. The assignment of the remaining methyl groups is then derived automatically as a byproduct alongside the structure calculation of the complexes by the *N*MR^2^ protocol.

The *N*MR^2^ structure calculation was conducted following the already published protocol using intraligand and intermolecular NOE-derived distances as well as partial methyl assignments.^31,46^ The total computational time was ∼1 h. The *N*MR^2^ structures reported are those with the lowest CYANA target functions. The *N*MR^2^ method does not use a force field but employs a hard sphere repulsion model for the atoms as described in the program CYANA.^59^ The *N*MR^2^ structures were then refined in explicit water solvent using the software NAMD (v 2.10) using the OPLS-AA force field.^60−63^ Complexes were solvated in a rectangular TIP3P water box with a 13 Å solvent padding using VMD (v.19.2).^64^ The NOE restraints were enforced by means of the collective variables Colvars function of NAMD with a force constant of 30 kcal/mol. The charge of the system was neutralized by adding suitable counterions. A time step of 2 fs was used with all of the bonds being constrained by means of the SHAKE algorithm. The electrostatic interactions were calculated by means of the PME method using a grid density of 1 bin/Å^3^. The VdW interactions were not considered beyond 12 Å after being gradually switched off starting from a distance of 10 Å. The temperature was controlled by coupling the system to a heat bath at 298 K. The pressure was kept at 1 atm by coupling the system to a pressure bath by means of the Berendsen barostat. The system was minimized for 2000 steps, followed by a 300 ps equilibration run and minimized again. During the equilibration run in addition to the NOE restraints, the protein backbone was restrained with a harmonic potential defined by a force constant of 6 kcal/mol, while the side chains were free.

#### BRD4 BD2

Interaction between the BRD4 BD2 and iBET762 was in the slow exchange regime on the NMR chemical shift time scale in agreement with the reported 16 nM affinity (Supplementary Figure S6).^49^ For the BRD4 BD2-iBET-762 complex, we measured 6 intraligand and 39 intermolecular NOEs (Supplementary Table S3). NOE build-up curves showing a slight deviation from the two-spin build-up curve model were used with a large tolerance for the upper limit restraint of 5.5 Å when a single ligand proton was involved in the NOE and 6.5 Å when two methyl groups were involved in the NOE, and no lower limit was applied. NOE build-up curves exhibiting a poor fit, due to spectral noise or strong spin diffusion, were discarded. In addition, in some cases, anti-NOE restraints were used. While several reasons can cause a NOE cross peak to disappear, such as dynamics-induced line broadening or spectral artifacts, we followed conservative guidelines to make use of absent NOEs. A missing NOE cross peak is interpreted as anti-NOE only if both protons involved exhibit visible NOE cross-peaks with other partners. The absence of a NOE can therefore unambiguously be attributed to a large distance between the two nuclei, and other effects can be excluded. The anti-NOEs used here are similar to those previously reported, except that we implemented them more conservatively, since we ensured that the anti-NOEs stem from protons that exhibit conventional NOEs and the corresponding lower limit restraints were reduced.^51^ The anti-NOEs were converted to 3 and 3.6 Å lower limit distance restraints when one methyl or two methyl groups were involved, respectively. Anti-NOEs contributed to an additional 37 lower limits restraints. Protein methyl—methyl distances were also observed in the half-filtered NOE experiments, and the pairing of the prochiral methyl groups of the valine and leucine amino acid residues could be derived thereof. *N*MR^2^ structure calculations were performed using intraligand- and intermolecular-derived distances, anti-NOEs distance restraints, and prochiral methyl groups pairing information. The total *N*MR^2^ structure calculation time was ∼15 min on a single machine (MacBook pro 2.7 GHz Intel Core i7), and the complex was refined similarly to the TRIM24 BD compound **1** complex.

## Supporting Information Available

Structures of compound **1** and IACS-9571 bound to TRIM24, comparison of iBET-762 bound to BRD4 BD2 and BRD2 BD2, backbone dihedral angles of the TRIM24 ZA loop, TRIM24 ZA-loop multiple sequence alignments, QC data for compound **1** and iBET-762, titrations of TRIM24 and BRD4 BD2 bromodomains followed by 2D NMR, constant time [^13^C,^1^H] HSQC of TRIM24 bound to compound **1**, predicted druggability scores and distance restraints used in the structure calculations (PDF)
Molecular formula strings (CSV)
Full wwPDB NMR structure validation report for 7AQT (PDF)
Full wwPDB NMR structure validation report D_1292111532 (PDF)

## Accession Codes

Structures and resonance assignments generated by the *N*MR^2^ algorithm for BRD4 BD2 in complex with iBET-762 and TRIM24 BD in complex with compound **1** have been deposited in the PDB/BMRB with accession codes 7AQT/34566 and 7B9X/34583, respectively.

## Abbreviations Used

ESI-MS: electrospray ionization mass spectrometry
HEPES: 2-[4-(2-hydroxyethyl)piperazin-1-yl]ethanesulfonic acid
IPTG: isopropyl-β-thiogalactoside
*N*MR^2^: NMR Molecular Replacement
NOE: nuclear Overhauser effect
OD: optical density
PAGE: polyacrylamide gel electrophoresis
SAR: structure–activity relationship
SBDD: structure-based drug discovery
SDS: sodium dodecyl sulfate
TCEP: tris(2-carboxyethyl)phosphine hydrochloride
TEV: tobacco etch virus
TRIS: tris(hydroxymethyl)aminomethane
