# Supplementary material for: NMR Molecular Replacement Provides New Insights into Binding Modes to Bromodomains of BRD4 and TRIM24
Source: J Med Chem. 2022 Mar 31;65(7):5565–74. doi: 10.1021/acs.jmedchem.1c01703 (PMC9017284; doi:10.1021/acs.jmedchem.1c01703)
Supplement: Supplementary file 1 — jm1c01703_si_001.pdf [file jm1c01703_si_001.pdf]

Supporting Information

NMR Molecular Replacement provides new insights into binding modes to bromodomains of BRD4 and TRIM24

Felix Torres‡ [1], Reto Walser‡ [2,3], Janina Kaderli [1], Emanuele Rossi [1], Romel Bobby [2,4], Martin J. Packer [5], Sunil Sarda [2], Graeme Walker [6], James R. Hitchin [6,7], Alexander G. Milbradt [2] and Julien Orts* [1,8]*

[1] ETH, Swiss Federal Institute of Technology, Laboratory of Physical Chemistry, HCI F217, Vladimir-Prelog-Weg 2, 8093 Zürich, Switzerland.

[2] BioPharmaceuticals R&D, AstraZeneca, Cambridge, CB4 0WG, United Kingdom

[3] present address: Astex Pharmaceuticals, 436 Cambridge Science Park, Milton Road, Cambridge, CB4 0QA, United Kingdom

[4] present address: Roche Pharma Research and Early Development, pRED Informatics, Roche Innovation Center Basel, F. Hoffmann-La Roche Ltd, Grenzacherstrasse 124, 4070 Basel, Switzerland

[5] Oncology R&D, AstraZeneca, Cambridge, CB4 0WG, United Kingdom

[6] Drug Discovery Unit, Cancer Research UK Manchester Institute, Alderley Park, Macclesfield, SK10 4TG, United Kingdom

[7] present address: Charnwood Molecular Ltd, BioCity, Nottingham, NG1 1GF, United Kingdom

[8] University of Vienna, Department of Pharmaceutical Sciences, Althanstrasse 14, A-1090 Vienna, Austria

* julien.orts@phys.chem.ethz.ch , alex.milbradt@astrazeneca.com

TABLE OF CONTENTS

Figure S1: Structures of compound 1 and IACS-9571 bound to TRIM24 ....S3

Figure S2: Comparison of iBET-762 bound to BRD4 BD2 and BRD2 BD2....................................S4

Figure S3: Backbone dihedral angles of the TRIM24 ZA-loop and TRIM24 ZA-loop multiple sequence alignments S5

Figure S4: QC data for iBET-762 ....S6

Figure S5: QC data for compound 1 ....S8

Figure S6: Titrations of TRIM24 and BRD4 BD2 bromodomains followed by 2D-NMR S9

Figure S7: Constant time [^{13}C,^{1}H] HSQC of TRIM24 bound to compound 1..................................S11

Table S1: Predicted druggability scores...S12

Table S2: Distance restraints used in the structure calculation of TRIM24 bound to compound 1...S13

Table S3: Distance restraints used in the structure calculation of BRD4 BD2 bound to iBET-762..S15

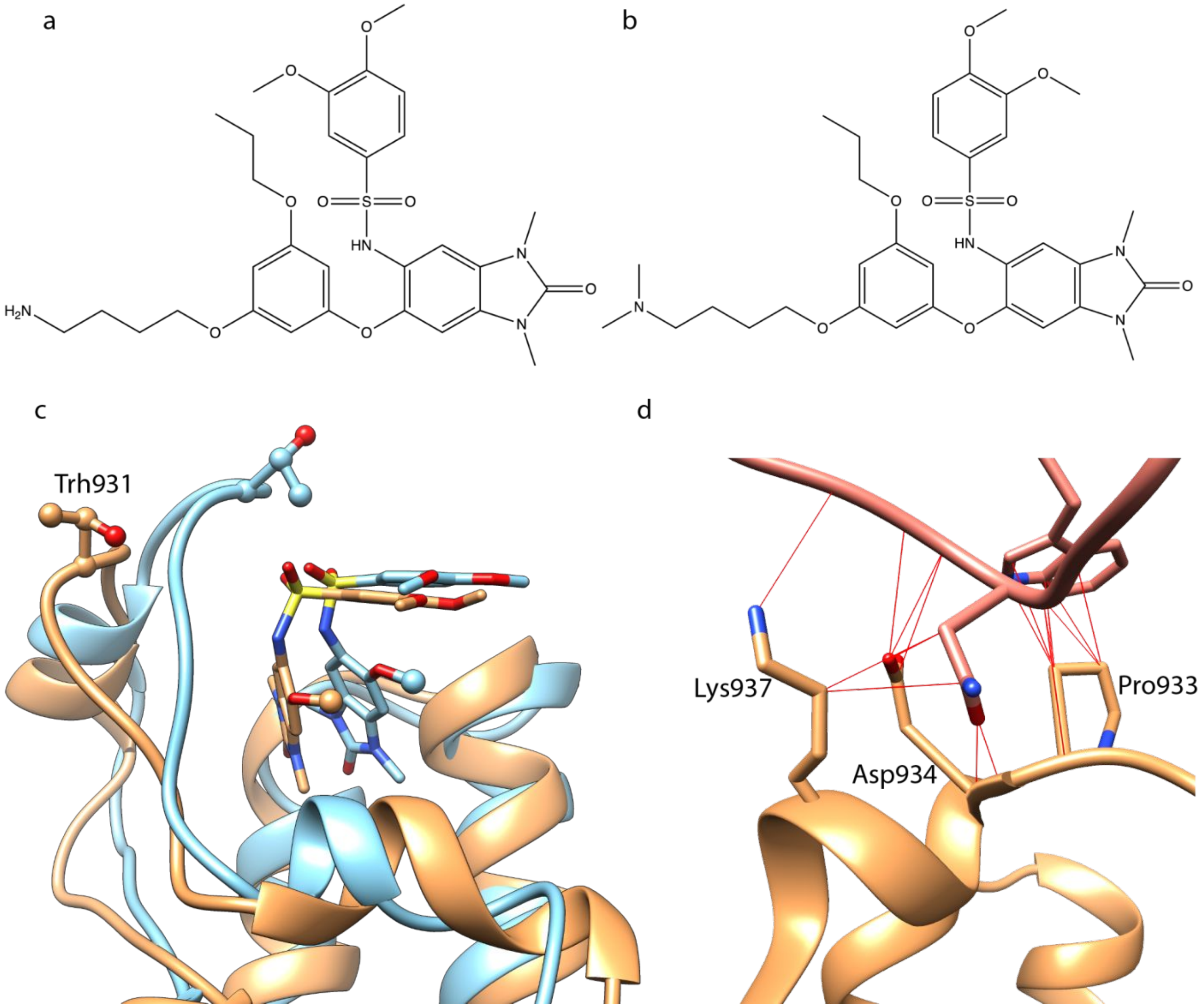

***Figure S1**: Structures of compound 1 and IACS-9571 bound to TRIM24*

a) 2D representation of compound 1. b) 2D representation of IACS-9571. c) Superposition of the TRIM24—compound 1 complex structure derived by *N*MR^2 (PDB code 7B9X) and TRIM24—IACS-9571 complex structure derived by X-ray crystallography (PDB code 4YC9). The protein ribbons are depicted in blue for the *N*MR^2 structure and orange for the X-ray crystallography structure. The aromatic moieties containing the aliphatic chains, ending with an ammonium group and a dimethylamino group for compound 1 and IACS-9571 respectively, are not shown for clarity

and replaced by a sphere. d) Crystal contacts between the ZA-loop of TRIM24 and a neighbor contacting molecule present in the crystallographic structure (PDB code 4YC9).

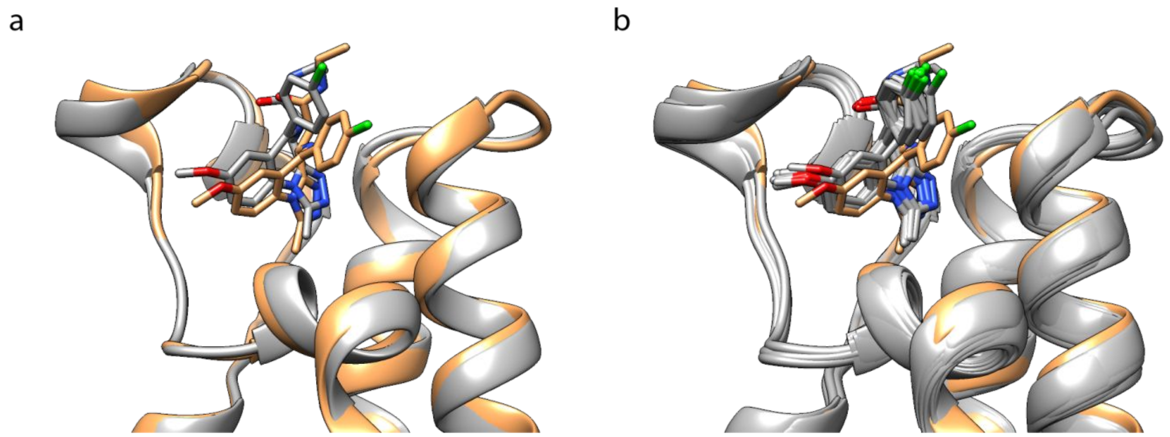

Figure S2: Comparison of iBET-762 bound to BRD4 BD2 and BRD2 BD2

a) Ribbon representation of the *N*MR^2 structure of BRD4 BD2 in complex with iBET-762 (PDB code 7AQT) superimposed to the X-ray structure of BRD2 BD2 in complex with iBET-762 (PDB code 5DFC). b) Same as in a) showing the *N*MR^2 structure bundle containing the ten lowest target function structures.

a)

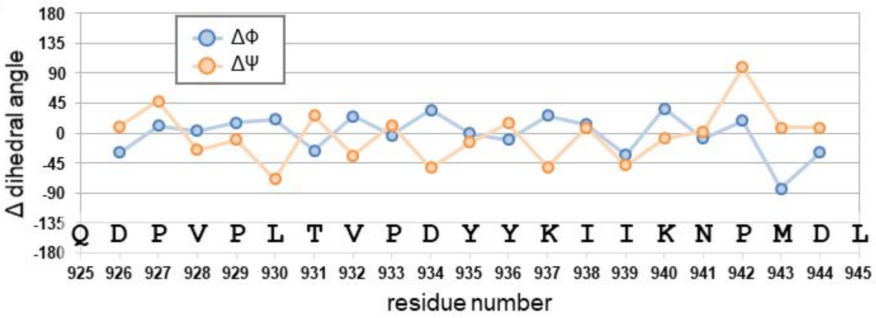

b)

```
           -925   -930        -935   -940   -945
TRIM24   QDPVPL--T-VPDYYKIIKNPMDL--       1.3
BRPF1    SEPVPLS--EVPDYLDHIKKPMDF--       2.1
BRD1     AQPVSLK--EVPDYLDHIKHPMDFAT       12
BRPF3    AEPVNLS--EVPDYLEFISKPMDFS-       27
BAZ2B    LLPVNLK--LVPGYKKVIKKPMDFS-       400
TAF1_2   HHPVNK--KFVPDYYKVIVNPMDL--       1800
BRD4_1   QQPVDAVKLNLPDYYKIIKTPMDM--       >10000
BRD4_2   YKPVDVEALGLHDYCDIIKHPMDM--       >10000
```

(Column heading printed at the right: K_d /nM)

***Figure S3**: Backbone dihedral angles of the TRIM24 ZA-loop and TRIM24 ZA-loop multiple sequence alignments*

a) Differences in backbone dihedral angles between the X-ray and *N*MR[2] structures of TRIM24 bromodomain and compound 1 (*N*MR[2]; PDB code 7B9X) or its (N,N)-dimethylated analogue IACS-9571 (X-ray; PDB code 4CY9) show that the loop closure of the ZA-loop is brought about by hinging motions of Leu930 at the N-terminus and Pro942 at the C-terminus of the loop. b) The bromodomains with a measurable affinity against IACS-9571 identified in a bromoscan[1] have a characteristic Leu at position 930 (numbering according to TRIM24 bromodomain). The bromodomains with the highest affinity have a PL-motif, other bromodomains with an appreciable

affinity against IACS-9571 have a [S/N]L-motif at that position. All other bromodomains are devoid of the characteristic Leu at position 930 and were inactive in the bromoscan.

a)

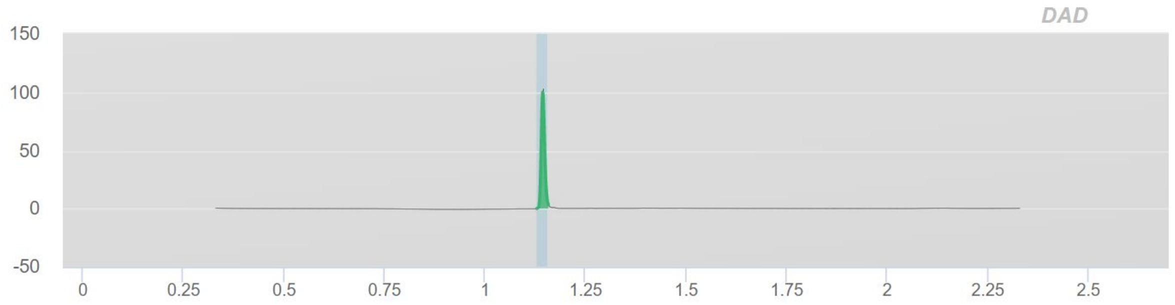

b)

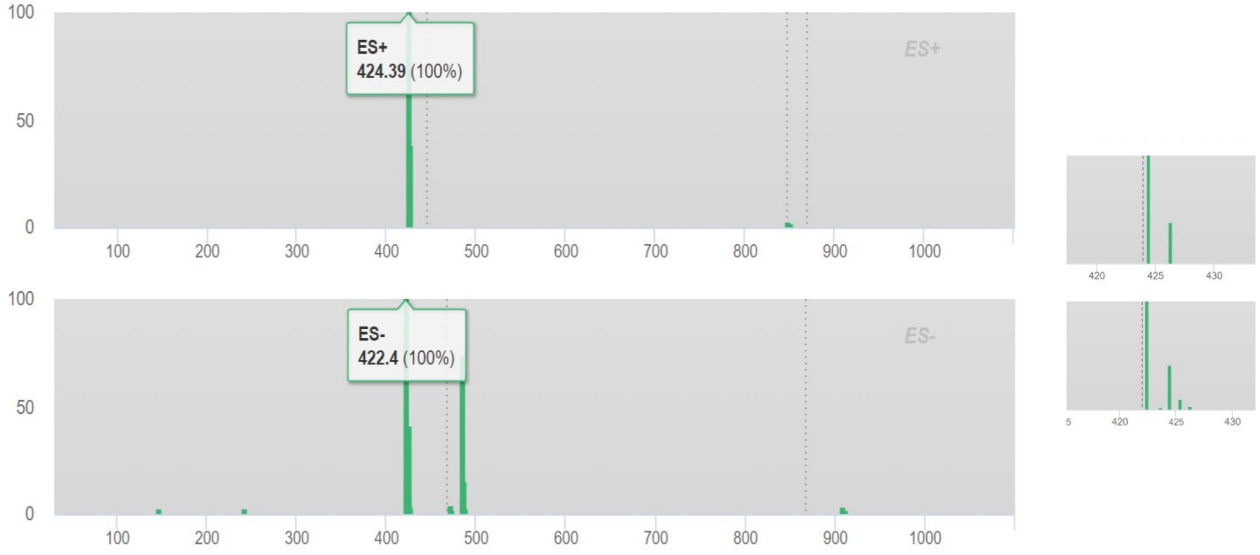

Figure S4: QC data for iBET-762

Purity determination of iBET-762 showing a) Diode Array Detection (UV) profile with b) corresponding electrospray mass spectra in positive (upper panel) and negative (lower panel) ion modes, respectively. The expected protonated and deprotonated molecular ions correspond to the

expected molecular mass of the compound (423 u), with the insets showing the expected mono-chlorinated isotopic configurations for each ion, respectively.

0.5 μL of 10 mM stock compound in dimethyl sulfoxide was diluted with 40 μL of a methanol:water mixture (50:50 v/v), with 1 μL of the resulting solution injected onto the LC-UV/MS system. Samples were analysed using gradient elution on a Waters BEH C_{18} 1.7 μm, 50 × 2.1 mm column (Waters Ltd., Wilmslow, UK) with a 3 μm (C_{18}) pre-column filter (Hichrom Ltd., Reading, UK) maintained at 40°C on a Waters Acquity UPLC system (Waters Ltd., Wilmslow, UK) flowing at 0.7 mL/min. The aqueous mobile phase (solvent A) was water and the organic solvent acetonitrile (solvent B), both containing 0.1% ammonium hydroxide as the modifier. The initial mobile phase consisted of 3% solvent B, held for 0.2 min, before increasing to 95% over 1 min, at which it was held for a further 0.2 min, before returning to initial conditions for the remainder of the 2.3 min run time. The eluent was passed via a UV flow cell set to detect between 210-400 nm wavelengths via diode array detection, onto the single quadrupole mass spectrometer (Waters SQD2, Waters Ltd., Wilmslow, UK). The Q1 full scan MS spectra were acquired between 100-1200 u mass ranges, toggling sequentially between positive and negative ion mode scans, with an inter-scan delay of 30 ms.

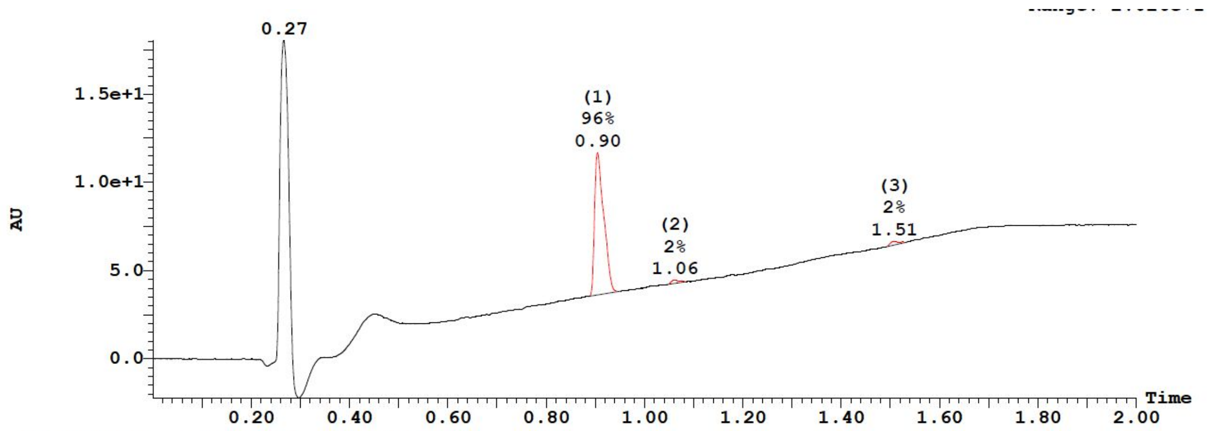

Peak Number	Time (mins)	Area %Total
1	0.90	95.72
2	1.06	1.98
3	1.51	2.30

Peak	Time (mins)
1	0.90

Peak	Time (mins)
1	0.90

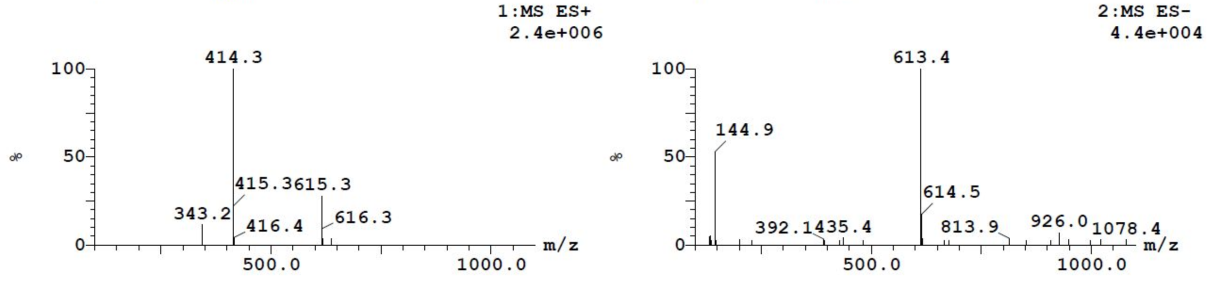

Figure S5: QC data for compound 1

Purity determination of compound 1. UV profile with corresponding electrospray mass spectra in positive (left panel) and negative (right panel) ion modes, respectively. The main peak in the UV trace (peak number 1) indicates a purity >95%. The expected protonated and deprotonated molecular ions of peak number 1 correspond to the expected molecular mass of compound 1 (615 u).

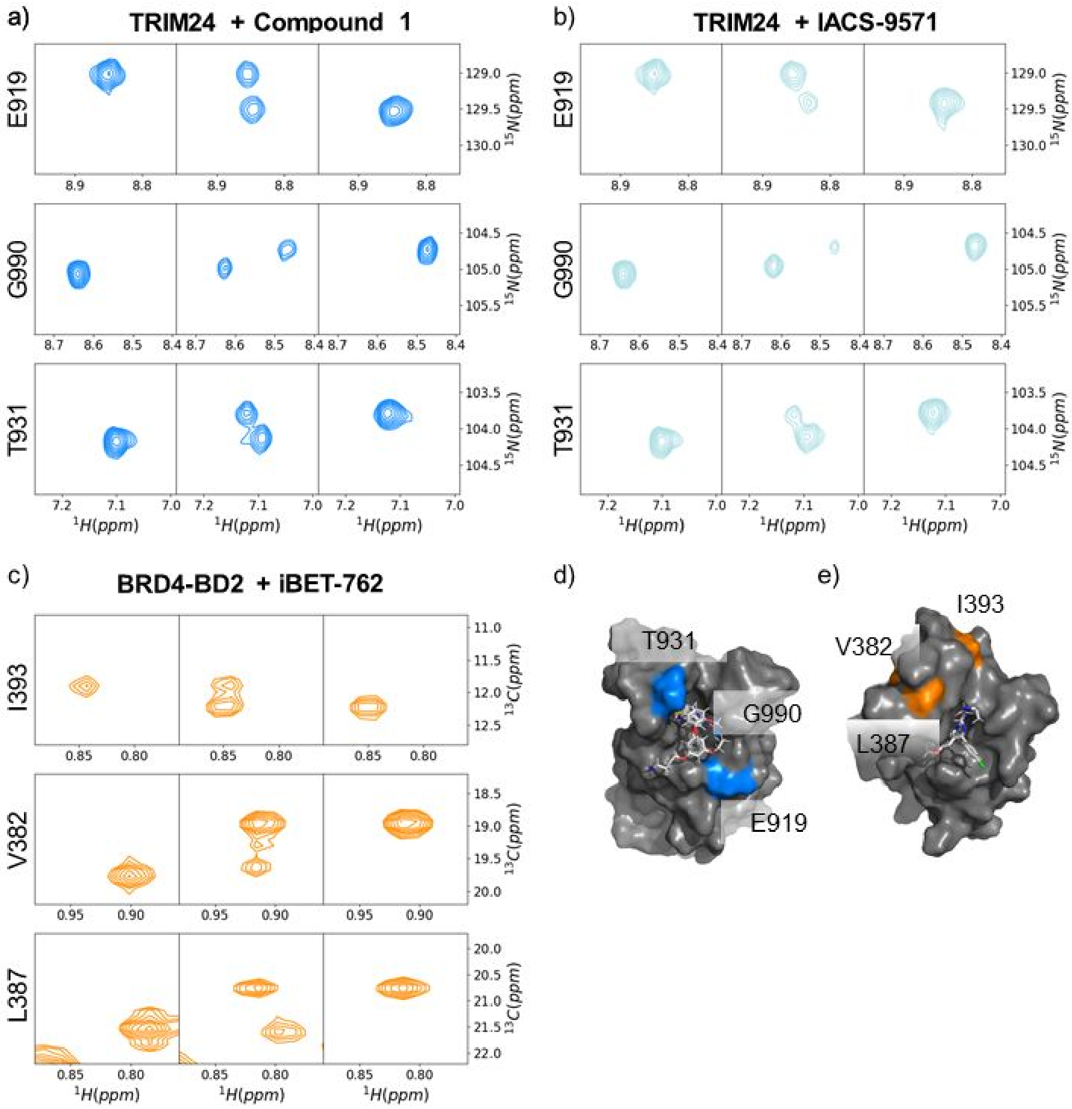

Figure S6: Titrations of TRIM24 and BRD4 BD2 bromodomains followed by 2D-NMR

a) TRIM24 titrated with compound 1 shows slow-exchange behavior typically observed for affinities in the sub-micromolar range. Chemical shift perturbations for three backbone amide peaks are followed at 0, 0.5 and 1 equivalent of compound 1 added to a sample of 0.1 mM ^{15}N-labelled TRIM24 (left, middle and right panel, respectively). b) TRIM24 titrated with the nanomolar binder IACS-9571, a close analogue of compound 1, exhibits the same slow exchange chemical shift perturbation pattern. The same three peaks as in panel a) are followed at 0, 0.3 and

1 equivalent of IACS-9571 added to a sample of 0.1 mM ^{15}N-labelled TRIM24. c) BRD4 BD2 titrated with iBET-762 shows slow-exchange behavior typical for sub-micromolar affinity interactions. Three peaks are followed at 0, 0.5 and 1 equivalent of iBET-762 added to a sample of 0.1 mM selectively methyl-labelled BRD4-BD2 at Val, Leu and Ile-δ1. d) Highlighting of residues followed in the titrations in panels a)-c) of TRIM24 (left; PDB code 7B9X) and BRD4-BD2 (right; PDB code 7AQT) on the structures solved in this publication.

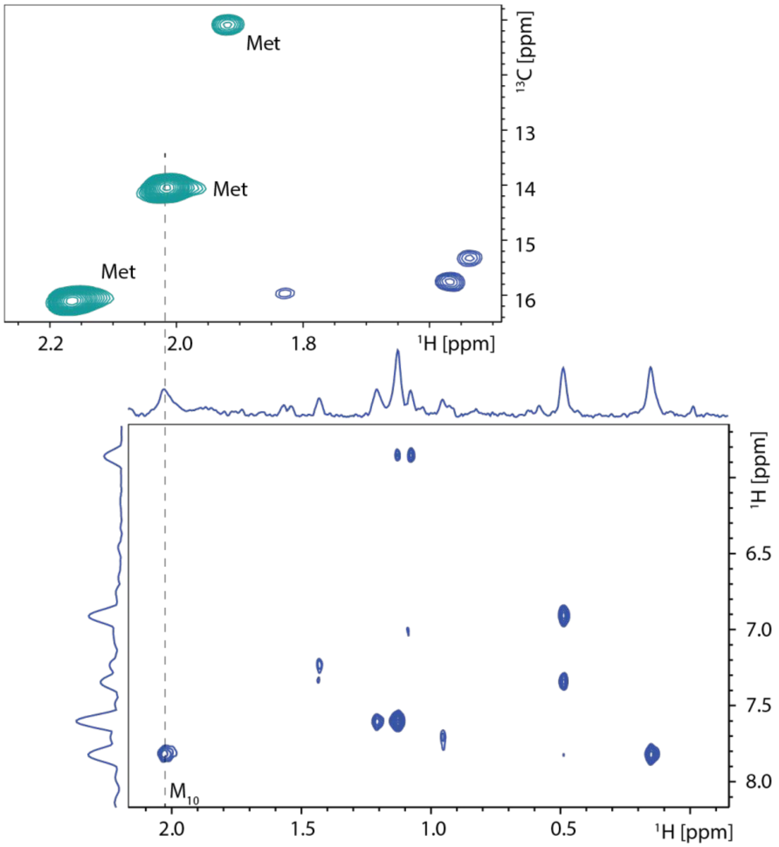

***Figure S7**: Constant time [^{13}C,^{1}H] HSQC of TRIM24 bound to compound 1*

Methionine region of a constant-time [^{13}C,^{1}H]-HSQC spectrum and the methyl-aromatic region of a [^{15}N,^{13}C]-filtered [^{1}H,^{1}H]-NOESY spectrum of TRIM24 (1.1 mM) with compound 1 (1 equivalent) measured on an 800 MHz spectrometer at 100 ms mixing time.

Table S1: Predicted druggability scores

Druggability scores as predicted by the Sitemap algorithm[2] as implemented in Schroedinger Maestro.[3] All parameters in this table were calculated in analogy to Vidler et al..[4]

PDB	Site score	Size	Dscore	Volume [Å^3]	Comment
4CY9	0.80	43	0.84	109	X-ray structure of IACS-9571 bound to TRIM24 BD
3O33	0.79	42	0.82	103	X-ray structure of apo TRIM24 BD
3MXF	0.87	51	0.91	136	X-ray structure of BRD4 BD1 bound to JQ1
7B9X	0.91	63	0.95	169	*N*MR[2] structure of compound 1 bound to TRIM24 BD

Table S2: *Distance restraints used in the structure calculation of TRIM24 bound to compound 1*

Intermolecular ligand—TRIM24 distances derived from the 2D-[^{13}C,^{15}N]-filtered-[^{1}H,^{1}H]-NOESY spectra series used to calculate the *N*MR^2 structure (PDB code 7B9X).

**Ligand**[a]	**Protein**[a]	**Lower limit distance [Å]**	**Upper limit distance [Å]**
H1	M7	2.8	4.0
H1	M8*	3.5	4.7
H2	M3	3.6	4.8
H5	M1	3.8	5.0
H5	M2	2.9	4.1
Q12	M1	3.3	4.8
Q12	M2	3.5	4.9
Q12	M3	4.2	5.6
Q12	M4	3.3	4.8
Q13	M10*	4.2	5.7
Q13	M2	3.6	5.0
Q13	M3	3.2	4.6
Q13	M4	3.7	5.2
Q13	M9	3.2	4.6
Q17	M5	4.0	5.4
Q17	M6	3.0	4.3
Q17	M6	3.2	4.4
Q17	M6	3.2	4.4
Q17	M7	3.4	4.6
Q17	M7	3.4	4.6
Q18	M5	4.3	5.7
Q18	M6	3.6	5.0

Q18	M7	4.0	5.4
Q9	M8*	1.9	7.6

[a] Ligand proton assignments follow the naming conventions in Figure 1a in the main manuscript and protein methyl assignments are unknown and named as M1-10. (*) M8 and M10 correspond to Thr931 and Met920, respectively.

Table S3: *Distance restraints used in the structure calculation of BRD4 BD2 bound to iBET-762*

Intermolecular ligand—BRD4 BD2 distances derived from the 2D-[^{13}C,^{15}N]-filtered-[^{1}H,^{1}H]-NOESY spectra series used to calculate the *N*MR^2 structure (PDB code 7AQT).

**Ligand[a]**	**Protein[a]**	**Lower limit distance [Å]**	**Upper limit distance [Å]**
QB	M8	2.9	4.3
QB	M5	4.0	5.5
QB	M6	4.3	5.9
QB	M4	0	6.5
Q15	M7	2.3	3.7
Q15	M6	3.9	5.3
Q15	M2	2.3	3.7
Q15	M1	2.6	5.5
Q15	M5	3.2	6.1
Q15	M3	0	6.5
Q15	M4	0	6.5
Q6	M6	2.6	5.5
Q6	M3	2.6	5.5
Q6	M7	0	6.5
Q6	M1	0	6.5
H5	M5	1.6	4.0
H5	M8	1.6	5.2
H5	M7	1.9	5.5

H5	M6	1.6	5.2
H5	M4	0	5.5
H5	M3	0	5.5
H5	M1	0	5.5
H2	M3	2.5	3.7
H2	M6	2.4	3.6
H2	M2	0	5.5
H19	M3	2.4	3.6
H19	M7	1.9	4.3
H19	M6	0	5.5
H19	M2	0	5.5
H19	M1	0	5.5
H1	M3	2.2	3.4
H1	M7	2.3	3.5
H1	M6	2.6	5.0
H1	M1	0	5.5
H4	M8	1.9	3.1
H4	M4	0	5.5
H4	M5	0	5.5
H9	M7	1.9	4.4
H8	M7	1.9	4.3

[a] Ligand proton assignments follow the conventions in Figure 3 in the main manuscript and protein methyl assignments are unknown and named as M1-8.

REFERENCES

1. Palmer, W. S.; Poncet-Montange, G.; Liu, G.; Petrocchi, A.; Reyna, N.; Subramanian, G.; Theroff, J.; Yau, A.; Kost-Alimova, M.; Bardenhagen, J. P.; Leo, E.; Shepard, H. E.; Tieu, T. N.; Shi, X.; Zhan, Y.; Zhao, S.; Barton, M. C.; Draetta, G.; Toniatti, C.; Jones, P.; Geck Do, M.; Andersen, J. N. Structure-guided design of IACS-9571, a selective high-affinity dual TRIM24-BRPF1 bromodomain inhibitor. J Med Chem 2016, 59 (4), 1440-1454. DOI: 10.1021/acs.jmedchem.5b00405.
2. Halgren, T. A.; Identifying and characterizing binding sites and assessing druggability. J Chem Inf Model 2009, 49 (2), 377−389. DOI: 10.1021/ci800324m.
3. SiteMap 2.5; Schrödinger, LLC: 101 SW Main Street, Suite 1300, Portland, OR 97204, U.S.A.; http://www.schrodinger.com/.
4. Vidler, L. R.; Brown, N.; Knapp, S.; Hoelder, S. Druggability analysis and structural classification of bromodomain acetyl-lysine binding sites. J Med Chem 2012, 55 (17), 7346-7359. DOI: 10.1021/jm300346w.
