# Supplementary material for: NMR Molecular Replacement Provides New Insights into Binding Modes to Bromodomains of BRD4 and TRIM24
Source: J Med Chem. 2022 Mar 31;65(7):5565–74. doi: 10.1021/acs.jmedchem.1c01703 (PMC9017284; doi:10.1021/acs.jmedchem.1c01703)
Supplement: Supplementary file 3 — jm1c01703_si_003.pdf [file jm1c01703_si_003.pdf]

Full wwPDB NMR Structure Validation Report (i)

Nov 5, 2020 – 12:31 PM GMT

PDB ID	:	7AQT
Title	:	NMR2 structure of BRD4-BD2 in complex with iBET-762
Deposited on	:	2020-10-23

This is a Full wwPDB NMR Structure Validation Report.

This report is produced by the wwPDB biocuration pipeline after annotation of the structure.

We welcome your comments at *validation@mail.wwpdb.org*
A user guide is available at
https://www.wwpdb.org/validation/2017/NMRValidationReportHelp
with specific help available everywhere you see the (i) symbol.

CONFIDENTIAL VALIDATION REPORT

The following versions of software and data (see references (i)) were used in the production of this report:

Cyrange	:	Kirchner and Güntert (2011)
NmrClust	:	Kelley et al. (1996)
MolProbity	:	4.02b-467
Mogul	:	1.8.5 (274361), CSD as541be (2020)
buster-report	:	1.1.7 (2018)
Percentile statistics	:	20191225.v01 (using entries in the PDB archive December 25th 2019)
RCI	:	v_1n_11_5_13_A (Berjanski et al., 2005)
PANAV	:	Wang et al. (2010)
ShiftChecker	:	2.14.6
Ideal geometry (proteins)	:	Engh & Huber (2001)
Ideal geometry (DNA, RNA)	:	Parkinson et al. (1996)
Validation Pipeline (wwPDB-VP)	:	2.14.6

1 Overall quality at a glance (i)

The following experimental techniques were used to determine the structure:
SOLUTION NMR

The overall completeness of chemical shifts assignment is 1%.

Percentile scores (ranging between 0-100) for global validation metrics of the entry are shown in the following graphic. The table shows the number of entries on which the scores are based.

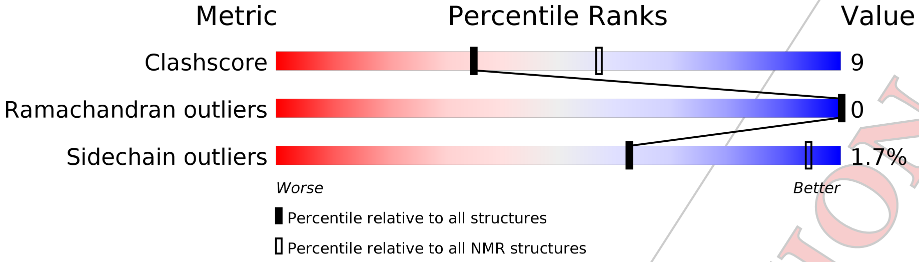

Metric	Whole archive (#Entries)	NMR archive (#Entries)
Clashscore	158937	12864
Ramachandran outliers	154571	11451
Sidechain outliers	154315	11428

The table below summarises the geometric issues observed across the polymeric chains and their fit to the experimental data. The red, orange, yellow and green segments indicate the fraction of residues that contain outliers for >=3, 2, 1 and 0 types of geometric quality criteria. A cyan segment indicates the fraction of residues that are not part of the well-defined cores, and a grey segment represents the fraction of residues that are not modelled. The numeric value for each fraction is indicated below the corresponding segment, with a dot representing fractions <=5%

Mol	Chain	Length	Quality of chain
1	A	109	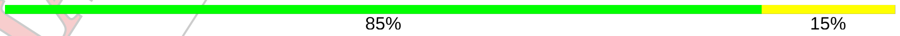

CONFIDENTIAL VALIDATION REPORT

2 Ensemble composition and analysis (i)

This entry contains 10 models. Model 7 is the overall representative, medoid model (most similar to other models).

The following residues are included in the computation of the global validation metrics.

Well-defined (core) protein residues			
**Well-defined core**	**Residue range (total)**	**Backbone RMSD (Å)**	**Medoid model**
1	A:4-A:112 (109)	0.00	7

Ill-defined regions of proteins are excluded from the global statistics.

Ligands and non-protein polymers are included in the analysis.

The models can be grouped into 1 clusters and 2 single-model clusters were found.

Cluster number	Models
1	1, 2, 5, 6, 7, 8, 9, 10
Single-model clusters	3; 4

CONFIDENTIAL VALIDATION REPORT

3 Entry composition (i)

There are 2 unique types of molecules in this entry. The entry contains 1811 atoms, of which 892 are hydrogens and 0 are deuteriums.

- Molecule 1 is a protein called Bromodomain-containing protein 4.

Mol	Chain	Residues	Atoms						Trace
			Total	C	H	N	O	S	
1	A	109	1759	569	870	149	160	11	0

- Molecule 2 is 2-[(4S)-6-(4-chlorophenyl)-8-methoxy-1-methyl-4H-[1,2,4]triazolo[4,3-a][1,4]benzodiazepin-4-yl]-N-ethylacetamide (three-letter code: EAM) (formula: $C_{22}H_{22}ClN_5O_2$).

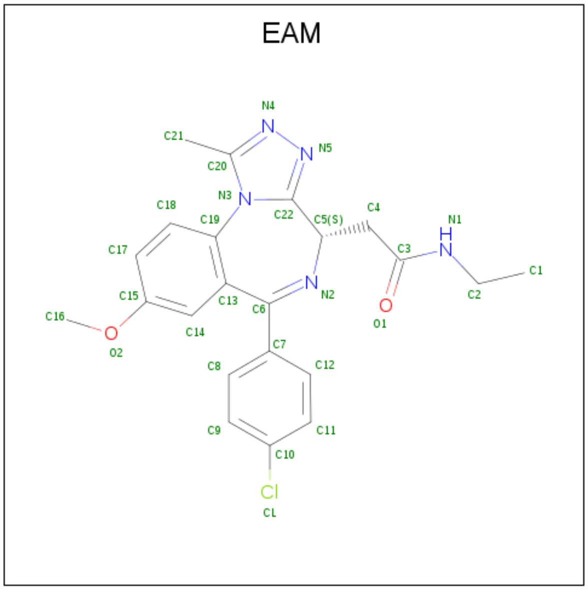

Mol	Chain	Residues	Atoms					
			Total	C	Cl	H	N	O
2	A	1	52	22	1	22	5	2

CONFIDENTIAL VALIDATION REPORT

4 Residue-property plots

4.1 Average score per residue in the NMR ensemble

These plots are provided for all protein, RNA, DNA and oligosaccharide chains in the entry. The first graphic is the same as shown in the summary in section 1 of this report. The second graphic shows the sequence where residues are colour-coded according to the number of geometric quality criteria for which they contain at least one outlier: green = 0, yellow = 1, orange = 2 and red = 3 or more. Stretches of 2 or more consecutive residues without any outliers are shown as green connectors. Residues which are classified as ill-defined in the NMR ensemble, are shown in cyan with an underline colour-coded according to the previous scheme. Residues which were present in the experimental sample, but not modelled in the final structure are shown in grey.

- Molecule 1: Bromodomain-containing protein 4

Chain A:

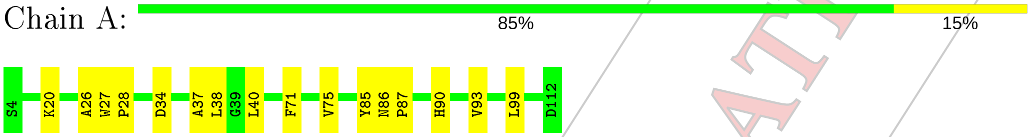

4.2 Scores per residue for each member of the ensemble

Colouring as in section 4.1 above.

4.2.1 Score per residue for model 1

- Molecule 1: Bromodomain-containing protein 4

Chain A:

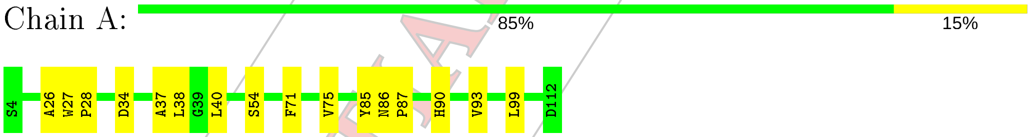

4.2.2 Score per residue for model 2

- Molecule 1: Bromodomain-containing protein 4

Chain A:

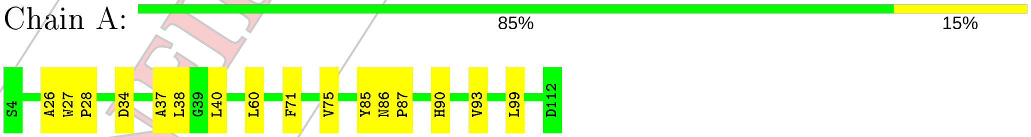

CONFIDENTIAL VALIDATION REPORT

4.2.3 Score per residue for model 3

• Molecule 1: Bromodomain-containing protein 4

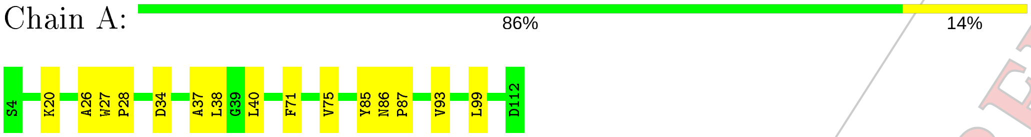

4.2.4 Score per residue for model 4

• Molecule 1: Bromodomain-containing protein 4

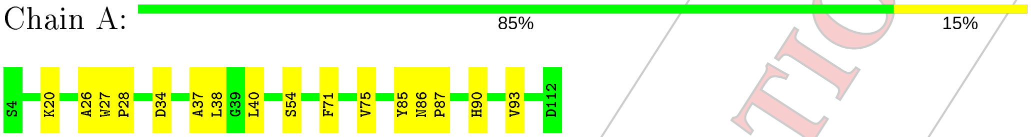

4.2.5 Score per residue for model 5

• Molecule 1: Bromodomain-containing protein 4

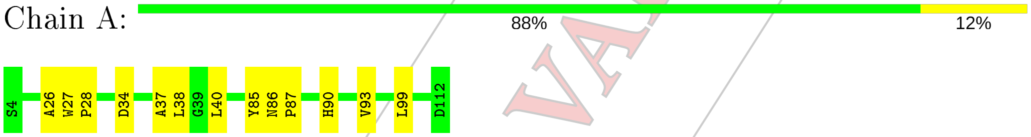

4.2.6 Score per residue for model 6

• Molecule 1: Bromodomain-containing protein 4

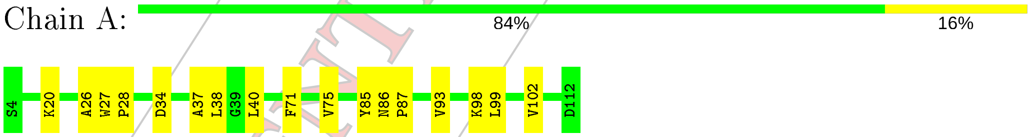

4.2.7 Score per residue for model 7 (medoid)

• Molecule 1: Bromodomain-containing protein 4

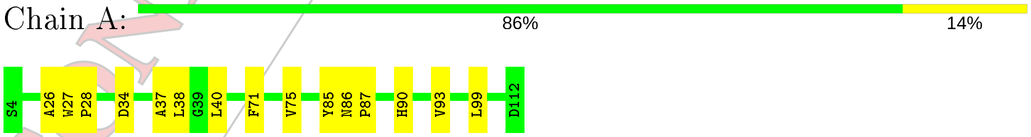

CONFIDENTIAL VALIDATION REPORT

4.2.8 Score per residue for model 8

- Molecule 1: Bromodomain-containing protein 4

Chain A:

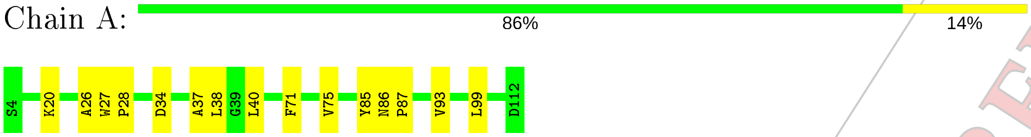

4.2.9 Score per residue for model 9

- Molecule 1: Bromodomain-containing protein 4

Chain A:

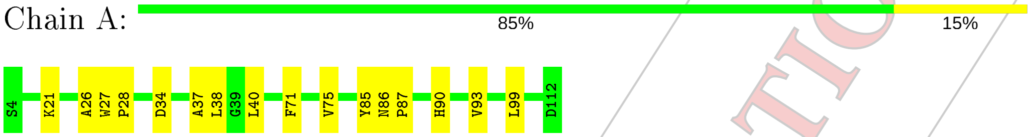

4.2.10 Score per residue for model 10

- Molecule 1: Bromodomain-containing protein 4

Chain A:

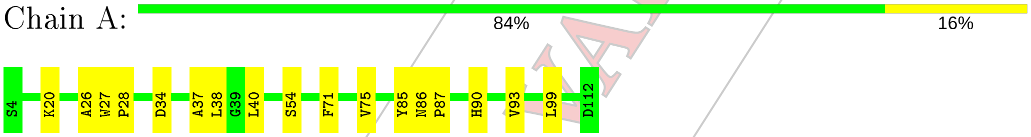

5 Refinement protocol and experimental data overview (i)

The models were refined using the following method: *NMR2*.

Of the ? calculated structures, 10 were deposited, based on the following criterion: *?*.

The following table shows the software used for structure solution, optimisation and refinement.

Software name	Classification	Version
CYANA	refinement	

The following table shows chemical shift validation statistics as aggregates over all chemical shift files. Detailed validation can be found in section 7 of this report.

Chemical shift file(s)	D_1292110224_cs_P1.cif.V1
Number of chemical shift lists	1
Total number of shifts	49
Number of shifts mapped to atoms	49
Number of unparsed shifts	0
Number of shifts with mapping errors	0
Number of shifts with mapping warnings	0
Assignment completeness (well-defined parts)	1%

No validations of the models with respect to experimental NMR restraints is performed at this time.

CONFIDENTIAL VALIDATION REPORT

6 Model quality (i)

6.1 Standard geometry (i)

Bond lengths and bond angles in the following residue types are not validated in this section: EAM

There are no covalent bond-length or bond-angle outliers.

There are no bond-length outliers.

There are no bond-angle outliers.

There are no chirality outliers.

There are no planarity outliers.

6.2 Too-close contacts (i)

In the following table, the Non-H and H(model) columns list the number of non-hydrogen atoms and hydrogen atoms in each chain respectively. The H(added) column lists the number of hydrogen atoms added and optimized by MolProbity. The Clashes column lists the number of clashes averaged over the ensemble.

Mol	Chain	Non-H	H(model)	H(added)	Clashes
1	A	889	870	869	14±1
2	A	30	22	22	5±0
All	All	9190	8920	8910	157

The all-atom clashscore is defined as the number of clashes found per 1000 atoms (including hydrogen atoms). The all-atom clashscore for this structure is 9.

All unique clashes are listed below, sorted by their clash magnitude.

Atom-1	Atom-2	Clash(Å)	Distance(Å)	Models	
				Worst	Total
1:A:27:TRP:N	1:A:28:PRO:HD2	0.88	1.84	4	10
1:A:40:LEU:HD21	2:A:201:EAM:C3	0.86	2.00	6	10
1:A:27:TRP:N	1:A:28:PRO:CD	0.81	2.44	9	10
1:A:26:ALA:HB2	1:A:99:LEU:HD13	0.66	1.66	9	9
1:A:40:LEU:HD21	2:A:201:EAM:O1	0.60	1.95	4	10
1:A:40:LEU:HD22	1:A:85:TYR:OH	0.56	2.01	8	10
1:A:26:ALA:C	1:A:28:PRO:HD2	0.55	2.21	3	10
1:A:27:TRP:CG	1:A:28:PRO:HD3	0.54	2.38	4	2
1:A:27:TRP:CD1	1:A:28:PRO:HD3	0.54	2.38	10	10
2:A:201:EAM:H4A	2:A:201:EAM:H1A	0.53	1.81	8	6
2:A:201:EAM:H1A	2:A:201:EAM:H4A	0.53	1.81	2	4

Continued on next page...

CONFIDENTIAL VALIDATION REPORT

Continued from previous page...

Atom-1	Atom-2	Clash(Å)	Distance(Å)	Models	
				Worst	Total
1:A:34:ASP:HB3	1:A:37:ALA:HB3	0.49	1.84	7	10
1:A:71:PHE:O	1:A:75:VAL:HG23	0.49	2.08	3	9
1:A:26:ALA:CB	1:A:99:LEU:HD13	0.48	2.39	5	2
1:A:87:PRO:O	1:A:93:VAL:HG21	0.47	2.09	7	10
1:A:60:LEU:HD11	1:A:71:PHE:CE1	0.47	2.44	2	1
1:A:98:LYS:O	1:A:102:VAL:HG23	0.45	2.12	6	1
1:A:34:ASP:O	1:A:38:LEU:HB2	0.44	2.13	3	10
2:A:201:EAM:C4	2:A:201:EAM:H1A	0.44	2.43	3	5
2:A:201:EAM:H1A	2:A:201:EAM:C4	0.44	2.43	1	5
1:A:86:ASN:OD1	2:A:201:EAM:N5	0.44	2.51	1	10
1:A:40:LEU:HD21	2:A:201:EAM:N1	0.40	2.30	1	3

6.3 Torsion angles (i)

6.3.1 Protein backbone (i)

In the following table, the Percentiles column shows the percent Ramachandran outliers of the chain as a percentile score with respect to all PDB entries followed by that with respect to all NMR entries. The Analysed column shows the number of residues for which the backbone conformation was analysed and the total number of residues.

Mol	Chain	Analysed	Favoured	Allowed	Outliers	Percentiles	
1	A	107/109 (98%)	102±0 (95±0%)	5±0 (5±0%)	0±0 (0±0%)	100	100
All	All	1070/1090 (98%)	1020 (95%)	50 (5%)	0 (0%)	100	100

There are no Ramachandran outliers.

6.3.2 Protein sidechains (i)

In the following table, the Percentiles column shows the percent sidechain outliers of the chain as a percentile score with respect to all PDB entries followed by that with respect to all NMR entries. The Analysed column shows the number of residues for which the sidechain conformation was analysed and the total number of residues.

Mol	Chain	Analysed	Rotameric	Outliers	Percentiles	
1	A	95/95 (100%)	93±1 (98±1%)	2±1 (2±1%)	62	94
All	All	950/950 (100%)	934 (98%)	16 (2%)	62	94

All 4 unique residues with a non-rotameric sidechain are listed below. They are sorted by the

CONFIDENTIAL VALIDATION REPORT

frequency of occurrence in the ensemble.

Mol	Chain	Res	Type	Models (Total)
1	A	90	HIS	7
1	A	20	LYS	5
1	A	54	SER	3
1	A	21	LYS	1

6.3.3 RNA (i)

There are no RNA molecules in this entry.

6.4 Non-standard residues in protein, DNA, RNA chains (i)

There are no non-standard protein/DNA/RNA residues in this entry.

6.5 Carbohydrates (i)

There are no monosaccharides in this entry.

6.6 Ligand geometry (i)

1 ligand is modelled in this entry.

In the following table, the Counts columns list the number of bonds for which Mogul statistics could be retrieved, the number of bonds that are observed in the model and the number of bonds that are defined in the chemical component dictionary. The Link column lists molecule types, if any, to which the group is linked. The Z score for a bond length is the number of standard deviations the observed value is removed from the expected value. A bond length with $|Z| > 2$ is considered an outlier worth inspection. RMSZ is the average root-mean-square of all Z scores of the bond lengths.

Mol	Type	Chain	Res	Link	Bond lengths		
					Counts	RMSZ	#Z>2
2	EAM	A	201	-	30,33,33	1.90±0.00	2±0 (6±0%)

In the following table, the Counts columns list the number of angles for which Mogul statistics could be retrieved, the number of angles that are observed in the model and the number of angles that are defined in the chemical component dictionary. The Link column lists molecule types, if any, to which the group is linked. The Z score for a bond angle is the number of standard deviations the observed value is removed from the expected value. A bond angle with $|Z| > 2$ is considered an outlier worth inspection. RMSZ is the average root-mean-square of all Z scores of the bond angles.

CONFIDENTIAL VALIDATION REPORT

Mol	Type	Chain	Res	Link	Bond angles		
					Counts	RMSZ	#Z>2
2	EAM	A	201	-	34,47,47	2.04±0.00	1±0 (2±0%)

In the following table, the Chirals column lists the number of chiral outliers, the number of chiral centers analysed, the number of these observed in the model and the number defined in the chemical component dictionary. Similar counts are reported in the Torsion and Rings columns. '-' means no outliers of that kind were identified.

Mol	Type	Chain	Res	Link	Chirals	Torsions	Rings
2	EAM	A	201	-	-	0±0,13,29,29	0±0,3,4,4

All unique bond outliers are listed below. They are sorted according to the Z-score of the worst occurrence in the ensemble.

Mol	Chain	Res	Type	Atoms	Z	Observed(Å)	Ideal(Å)	Models	
								Worst	Total
2	A	201	EAM	C13-C6	5.56	1.41	1.49	2	10
2	A	201	EAM	C7-C6	5.55	1.41	1.49	2	10

All unique angle outliers are listed below.

Mol	Chain	Res	Type	Atoms	Z	Observed(^{o})	Ideal(^{o})	Models	
								Worst	Total
2	A	201	EAM	C5-N2-C6	7.30	124.70	117.62	2	10

There are no chirality outliers.

There are no torsion outliers.

There are no ring outliers.

The following is a two-dimensional graphical depiction of Mogul quality analysis of bond lengths, bond angles, torsion angles, and ring geometry for all instances of the Ligand of Interest. In addition, ligands with molecular weight > 250 and outliers as shown on the validation Tables will also be included. For torsion angles, if less then 5% of the Mogul distribution of torsion angles is within 10 degrees of the torsion angle in question, then that torsion angle is considered an outlier. Any bond that is central to one or more torsion angles identified as an outlier by Mogul will be highlighted in the graph. For rings, the root-mean-square deviation (RMSD) between the ring in question and similar rings identified by Mogul is calculated over all ring torsion angles. If the average RMSD is greater than 60 degrees and the minimal RMSD between the ring in question and any Mogul-identified rings is also greater than 60 degrees, then that ring is considered an outlier. The outliers are highlighted in purple. The color gray indicates Mogul did not find sufficient equivalents in the CSD to analyse the geometry.

CONFIDENTIAL VALIDATION REPORT

Ligand EAM A 201

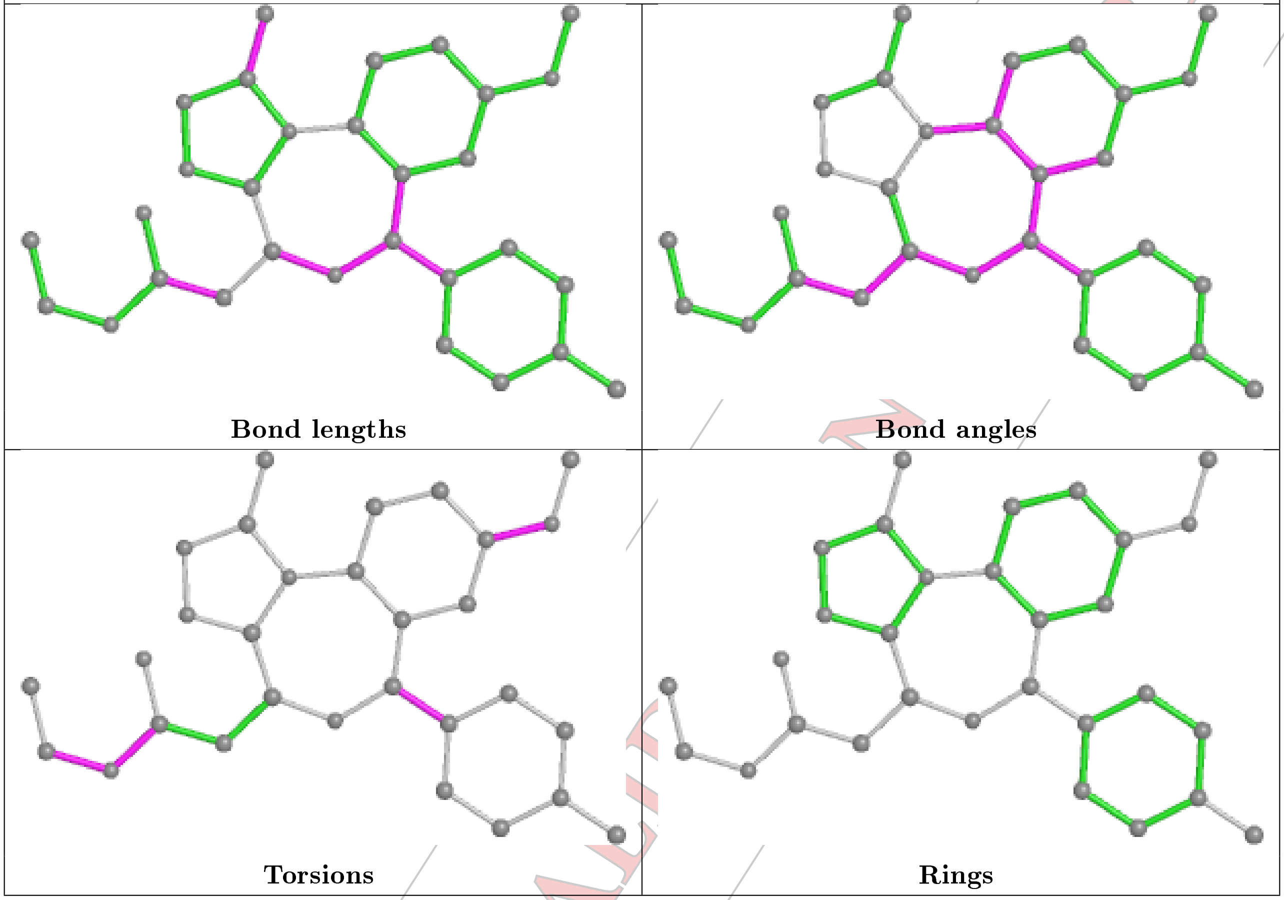

6.7 Other polymers (i)

There are no such molecules in this entry.

6.8 Polymer linkage issues (i)

There are no chain breaks in this entry.

CONFIDENTIAL VALIDATION REPORT

7 Chemical shift validation (i)

The completeness of assignment taking into account all chemical shift lists is 1% for the well-defined parts and 1% for the entire structure.

7.1 Chemical shift list 1

File name: D_1292110224_cs_P1.cif.V1

Chemical shift list name: *assigned_chem_shift_list*

7.1.1 Bookkeeping (i)

The following table shows the results of parsing the chemical shift list and reports the number of nuclei with statistically unusual chemical shifts.

Total number of shifts	49
Number of shifts mapped to atoms	49
Number of unparsed shifts	0
Number of shifts with mapping errors	0
Number of shifts with mapping warnings	0
Number of shift outliers (ShiftChecker)	0

7.1.2 Chemical shift referencing (i)

No chemical shift referencing corrections were calculated (not enough data).

7.1.3 Completeness of resonance assignments (i)

The following table shows the completeness of the chemical shift assignments for the well-defined regions of the structure. The overall completeness is 1%, i.e. 16 atoms were assigned a chemical shift out of a possible 1393. 4 out of 13 assigned methyl groups (LEU and VAL) were assigned stereospecifically.

	Total	^{1}H	^{13}C	^{15}N
Backbone	0/533 (0%)	0/212 (0%)	0/218 (0%)	0/103 (0%)
Sidechain	16/718 (2%)	8/428 (2%)	8/259 (3%)	0/31 (0%)
Aromatic	0/142 (0%)	0/76 (0%)	0/61 (0%)	0/5 (0%)
Overall	16/1393 (1%)	8/716 (1%)	8/538 (1%)	0/139 (0%)

The following table shows the completeness of the chemical shift assignments for the full structure. The overall completeness is 1%, i.e. 16 atoms were assigned a chemical shift out of a possible 1393. 4 out of 13 assigned methyl groups (LEU and VAL) were assigned stereospecifically.

CONFIDENTIAL VALIDATION REPORT

	Total	^{1}H	^{13}C	^{15}N
Backbone	0/533 (0%)	0/212 (0%)	0/218 (0%)	0/103 (0%)
Sidechain	16/718 (2%)	8/428 (2%)	8/259 (3%)	0/31 (0%)
Aromatic	0/142 (0%)	0/76 (0%)	0/61 (0%)	0/5 (0%)
Overall	16/1393 (1%)	8/716 (1%)	8/538 (1%)	0/139 (0%)

7.1.4 Statistically unusual chemical shifts ⓘ

There are no statistically unusual chemical shifts.

7.1.5 Random Coil Index (RCI) plots ⓘ

No *random coil index* (RCI) plot could be generated from the current chemical shift list (assigned_chem_shift_list). RCI is only applicable to proteins.

CONFIDENTIAL VALIDATION REPORT
