# Supplementary material for: NMR Molecular Replacement Provides New Insights into Binding Modes to Bromodomains of BRD4 and TRIM24
Source: J Med Chem. 2022 Mar 31;65(7):5565–74. doi: 10.1021/acs.jmedchem.1c01703 (PMC9017284; doi:10.1021/acs.jmedchem.1c01703)
Supplement: Supplementary file 4 — jm1c01703_si_004.pdf [file jm1c01703_si_004.pdf]

Preliminary Full wwPDB NMR Structure Validation Report (i)

Dec 14, 2020 – 10:43 pm GMT

Deposition ID : D_1292111532

This is a Preliminary Full wwPDB NMR Structure Validation Report.

This report is produced by the wwPDB Deposition System during initial deposition but before annotation of the structure.

We welcome your comments at *validation@mail.wwpdb.org*
A user guide is available at
https://www.wwpdb.org/validation/2017/NMRValidationReportHelp
with specific help available everywhere you see the (i) symbol.

The following versions of software and data (see references (i)) were used in the production of this report:

MolProbity	:	4.02b-467
Mogul	:	1.8.5 (274361), CSD as541be (2020)
Percentile statistics	:	20191225.v01 (using entries in the PDB archive December 25th 2019)
RCI	:	v_1n_11_5_13_A (Berjanski et al., 2005)
PANAV	:	Wang et al. (2010)
ShiftChecker	:	2.15.1
Ideal geometry (proteins)	:	Engh & Huber (2001)
Ideal geometry (DNA, RNA)	:	Parkinson et al. (1996)
Validation Pipeline (wwPDB-VP)	:	2.15.1

PRELIMINARY VALIDATION REPORT

1 Overall quality at a glance (i)

The following experimental techniques were used to determine the structure:
SOLUTION NMR

The overall completeness of chemical shifts assignment was not calculated.

Percentile scores (ranging between 0-100) for global validation metrics of the entry are shown in the following graphic. The table shows the number of entries on which the scores are based.

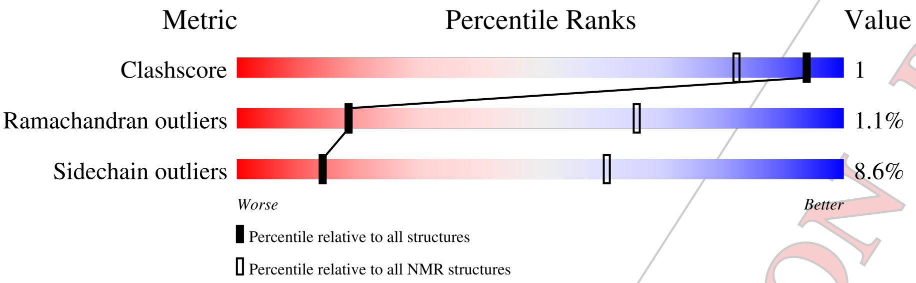

Metric	Whole archive (#Entries)	NMR archive (#Entries)
Clashscore	158937	12864
Ramachandran outliers	154571	11451
Sidechain outliers	154315	11428

The table below summarises the geometric issues observed across the polymeric chains and their fit to the experimental data. The red, orange, yellow and green segments indicate the fraction of residues that contain outliers for >=3, 2, 1 and 0 types of geometric quality criteria. A cyan segment indicates the fraction of residues that are not part of the well-defined cores, and a grey segment represents the fraction of residues that are not modelled. The numeric value for each fraction is indicated below the corresponding segment, with a dot representing fractions <=5%

Mol	Chain	Length	Quality of chain
1	A	106	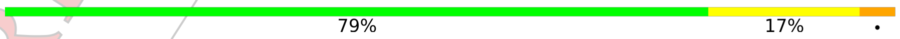

PRELIMINARY VALIDATION REPORT

2 Ensemble composition and analysis (i)

This entry contains 10 models. Model 2 is the overall representative, medoid model (most similar to other models).

The following residues are included in the computation of the global validation metrics.

Well-defined (core) protein residues			
**Well-defined core**	**Residue range (total)**	**Backbone RMSD (Å)**	**Medoid model**
1	A:901-A:1006 (106)	0.17	2

Ill-defined regions of proteins are excluded from the global statistics.

Ligands and non-protein polymers are included in the analysis.

The models can be grouped into 1 clusters and 3 single-model clusters were found.

Cluster number	Models
1	1, 2, 3, 4, 5, 6, 10
Single-model clusters	7; 8; 9

PRELIMINARY VALIDATION REPORT

3 Entry composition (i)

There are 2 unique types of molecules in this entry. The entry contains 1837 atoms, of which 911 are hydrogens and 0 are deuteriums.

- Molecule 1 is a protein called TRIM24-BD.

Mol	Chain	Residues	Atoms						Trace
			Total	C	H	N	O	S	
1	A	106	1755	573	872	138	166	6	0

- Molecule 2 is a ligand with the chemical component id JO2 but its atom names do not match the existing wwPDB Chemical Component Dictionary definition for JO2. Consequently no firm identification of ligand chemistry can be made. Once the structure is annotated then an identification and diagram will be given here.

Mol	Chain	Residues	Atoms					
			Total	C	H	N	O	S
2	A	1	82	30	39	4	8	1

PRELIMINARY VALIDATION REPORT

4 Residue-property plots (i)

4.1 Average score per residue in the NMR ensemble

These plots are provided for all protein, RNA, DNA and oligosaccharide chains in the entry. The first graphic is the same as shown in the summary in section 1 of this report. The second graphic shows the sequence where residues are colour-coded according to the number of geometric quality criteria for which they contain at least one outlier: green = 0, yellow = 1, orange = 2 and red = 3 or more. Stretches of 2 or more consecutive residues without any outliers are shown as green connectors. Residues which are classified as ill-defined in the NMR ensemble, are shown in cyan with an underline colour-coded according to the previous scheme. Residues which were present in the experimental sample, but not modelled in the final structure are shown in grey.

- Molecule 1: TRIM24-BD

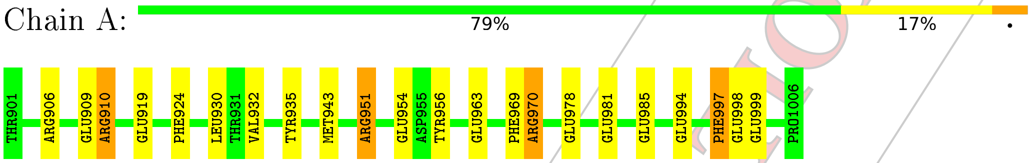

4.2 Scores per residue for each member of the ensemble

Colouring as in section 4.1 above.

4.2.1 Score per residue for model 1

- Molecule 1: TRIM24-BD

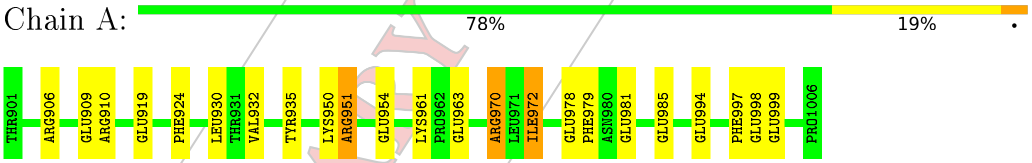

4.2.2 Score per residue for model 2 (medoid)

- Molecule 1: TRIM24-BD

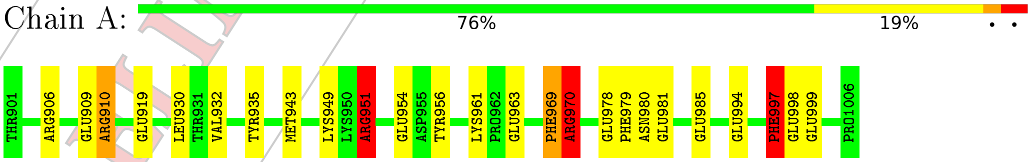

PRELIMINARY VALIDATION REPORT

4.2.3 Score per residue for model 3

- Molecule 1: TRIM24-BD

Chain A:

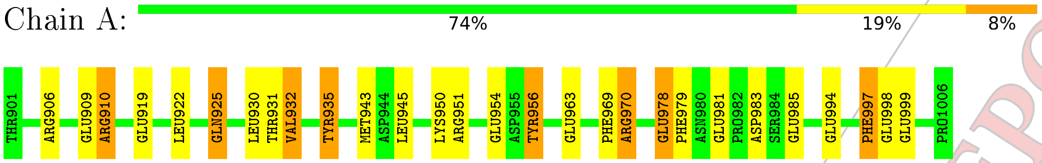

4.2.4 Score per residue for model 4

- Molecule 1: TRIM24-BD

Chain A:

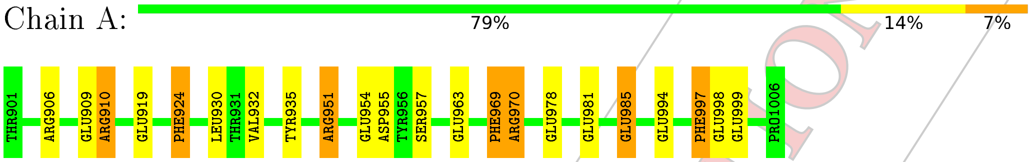

4.2.5 Score per residue for model 5

- Molecule 1: TRIM24-BD

Chain A:

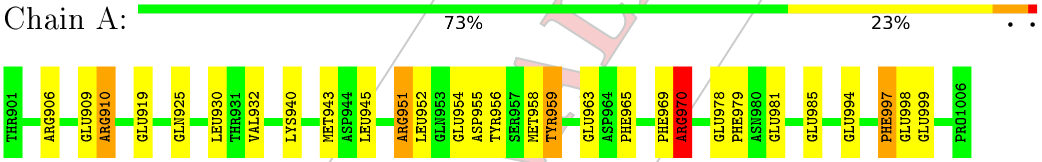

4.2.6 Score per residue for model 6

- Molecule 1: TRIM24-BD

Chain A:

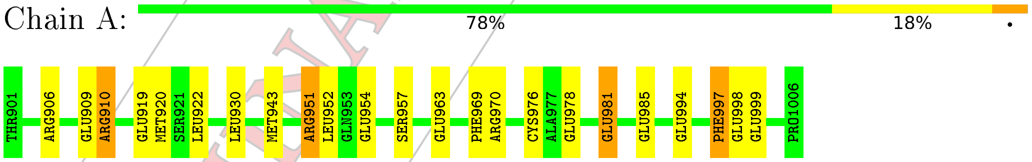

4.2.7 Score per residue for model 7

- Molecule 1: TRIM24-BD

Chain A:

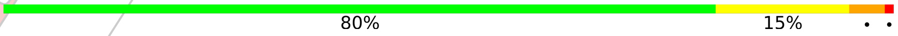

PRELIMINARY VALIDATION REPORT

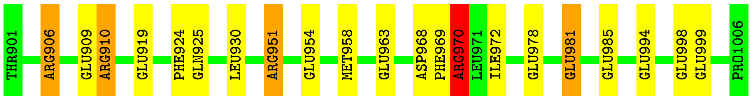

4.2.8 Score per residue for model 8

• Molecule 1: TRIM24-BD

Chain A:

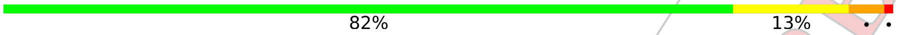

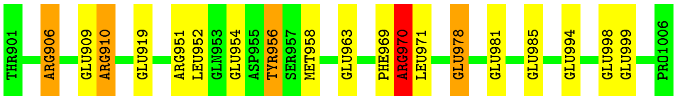

4.2.9 Score per residue for model 9

• Molecule 1: TRIM24-BD

Chain A:

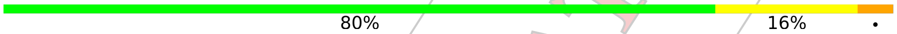

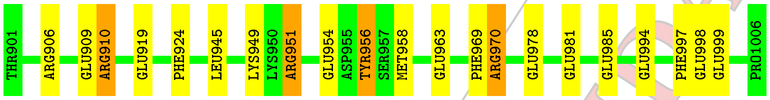

4.2.10 Score per residue for model 10

• Molecule 1: TRIM24-BD

Chain A:

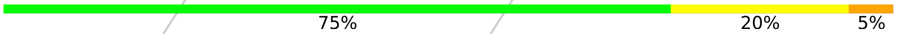

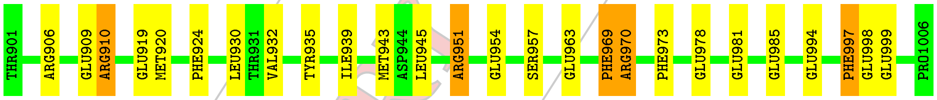

PRELIMINARY VALIDATION REPORT

5 Refinement protocol and experimental data overview (i)

Of the ? calculated structures, 10 were deposited, based on the following criterion: ?.

The following table shows the software used for structure solution, optimisation and refinement.

Software name	Classification	Version
.	refinement	
.	structure calculation	

The following table shows chemical shift validation statistics as aggregates over all chemical shift files. Detailed validation can be found in section 7 of this report.

Chemical shift file(s)	D_1292111532_cs_P1.cif.V11
Number of chemical shift lists	1
Total number of shifts	62
Number of shifts mapped to atoms	0
Number of unparsed shifts	62
Number of shifts with mapping errors	0
Number of shifts with mapping warnings	0
Assignment completeness (well-defined parts)	0%

PRELIMINARY VALIDATION REPORT

6 Model quality (i)

6.1 Standard geometry (i)

Bond lengths and bond angles in the following residue types are not validated in this section: JO2

The Z score for a bond length (or angle) is the number of standard deviations the observed value is removed from the expected value. A bond length (or angle) with $|Z| > 5$ is considered an outlier worth inspection. RMSZ is the (average) root-mean-square of all Z scores of the bond lengths (or angles).

Mol	Chain	Bond lengths		Bond angles	
		RMSZ	#Z>5	RMSZ	#Z>5
1	A	1.55±0.01	28±0/905 (3.1± 0.0%)	1.23±0.02	5±1/1224 (0.4± 0.1%)
All	All	1.55	279/9050 (3.1%)	1.23	45/12240 (0.4%)

Chiral center outliers are detected by calculating the chiral volume of a chiral center and verifying if the center is modelled as a planar moiety or with the opposite hand. A planarity outlier is detected by checking planarity of atoms in a peptide group, atoms in a mainchain group or atoms of a sidechain that are expected to be planar.

Mol	Chain	Chirality	Planarity
1	A	0.0±0.0	2.8±0.9
All	All	0	28

All unique bond outliers are listed below. They are sorted according to the Z-score of the worst occurrence in the ensemble.

Mol	Chain	Res	Type	Atoms	Z	Observed(Å)	Ideal(Å)	Models	
								Worst	Total
1	A	951	ARG	NE-CZ	9.84	1.45	1.33	4	10
1	A	970	ARG	NE-CZ	9.82	1.45	1.33	3	10
1	A	906	ARG	NE-CZ	9.52	1.45	1.33	3	10
1	A	910	ARG	NE-CZ	9.36	1.45	1.33	2	10
1	A	970	ARG	CZ-NH1	9.26	1.45	1.33	4	10
1	A	951	ARG	CZ-NH1	9.14	1.45	1.33	8	10
1	A	906	ARG	CZ-NH1	9.06	1.44	1.33	10	10
1	A	910	ARG	CZ-NH1	8.91	1.44	1.33	5	10
1	A	998	GLU	CD-OE1	5.78	1.32	1.25	3	10
1	A	999	GLU	CD-OE1	5.71	1.31	1.25	4	10
1	A	978	GLU	CD-OE1	5.68	1.31	1.25	1	10
1	A	985	GLU	CD-OE1	5.67	1.31	1.25	1	10
1	A	919	GLU	CD-OE1	5.66	1.31	1.25	8	10
1	A	954	GLU	CD-OE1	5.64	1.31	1.25	3	10

Continued on next page...

PRELIMINARY VALIDATION REPORT

Continued from previous page...

Mol	Chain	Res	Type	Atoms	Z	Observed(Å)	Ideal(Å)	Models	
								Worst	Total
1	A	954	GLU	CD-OE2	5.64	1.31	1.25	4	10
1	A	981	GLU	CD-OE1	5.63	1.31	1.25	3	10
1	A	978	GLU	CD-OE2	5.63	1.31	1.25	8	9
1	A	994	GLU	CD-OE1	5.63	1.31	1.25	7	10
1	A	985	GLU	CD-OE2	5.62	1.31	1.25	2	10
1	A	909	GLU	CD-OE1	5.61	1.31	1.25	6	10
1	A	994	GLU	CD-OE2	5.61	1.31	1.25	5	10
1	A	909	GLU	CD-OE2	5.61	1.31	1.25	7	10
1	A	999	GLU	CD-OE2	5.61	1.31	1.25	9	10
1	A	919	GLU	CD-OE2	5.61	1.31	1.25	9	10
1	A	981	GLU	CD-OE2	5.60	1.31	1.25	10	10
1	A	963	GLU	CD-OE2	5.60	1.31	1.25	9	10
1	A	963	GLU	CD-OE1	5.58	1.31	1.25	6	10
1	A	998	GLU	CD-OE2	5.58	1.31	1.25	8	10

All unique angle outliers are listed below. They are sorted according to the Z-score of the worst occurrence in the ensemble.

Mol	Chain	Res	Type	Atoms	Z	Observed(°)	Ideal(°)	Models	
								Worst	Total
1	A	970	ARG	CD-NE-CZ	-10.74	108.56	123.60	1	10
1	A	951	ARG	CD-NE-CZ	-10.08	109.49	123.60	2	9
1	A	906	ARG	CD-NE-CZ	-9.38	110.46	123.60	7	10
1	A	935	TYR	CB-CG-CD2	-7.27	116.64	121.00	3	3
1	A	910	ARG	CD-NE-CZ	-7.22	113.50	123.60	8	4
1	A	935	TYR	CB-CG-CD1	-6.40	117.16	121.00	4	3
1	A	956	TYR	CB-CG-CD1	-6.21	117.27	121.00	8	3
1	A	956	TYR	CB-CG-CD2	5.42	124.25	121.00	8	1
1	A	924	PHE	CB-CG-CD2	5.22	124.46	120.80	4	1
1	A	959	TYR	CB-CG-CD1	-5.03	117.98	121.00	5	1

There are no chirality outliers.

All unique planar outliers are listed below. They are sorted by the frequency of occurrence in the ensemble.

Mol	Chain	Res	Type	Group	Models (Total)
1	A	970	ARG	Sidechain	9
1	A	910	ARG	Sidechain	9
1	A	951	ARG	Sidechain	7
1	A	906	ARG	Sidechain	2
1	A	997	PHE	Sidechain	1

PRELIMINARY VALIDATION REPORT

6.2 Too-close contacts (i)

In the following table, the Non-H and H(model) columns list the number of non-hydrogen atoms and hydrogen atoms in each chain respectively. The H(added) column lists the number of hydrogen atoms added and optimized by MolProbity. The Clashes column lists the number of clashes averaged over the ensemble.

Mol	Chain	Non-H	H(model)	H(added)	Clashes
1	A	883	872	870	2±1
All	All	9260	9110	8700	22

The all-atom clashscore is defined as the number of clashes found per 1000 atoms (including hydrogen atoms). The all-atom clashscore for this structure is 1.

All unique clashes are listed below, sorted by their clash magnitude.

Atom-1	Atom-2	Clash(Å)	Distance(Å)	Models	
				Worst	Total
1:A:969:PHE:CE1	1:A:997:PHE:CZ	0.52	2.97	2	2
1:A:969:PHE:CZ	1:A:997:PHE:CZ	0.50	3.00	5	5
1:A:969:PHE:CE2	1:A:997:PHE:CZ	0.50	3.00	6	1
1:A:997:PHE:CD1	1:A:997:PHE:C	0.48	2.86	2	4
1:A:997:PHE:C	1:A:997:PHE:CD1	0.46	2.88	4	3
1:A:959:TYR:CD1	1:A:959:TYR:N	0.44	2.86	5	1
1:A:924:PHE:CZ	1:A:972:ILE:HG22	0.43	2.48	1	1
1:A:932:VAL:HG21	1:A:935:TYR:CD2	0.42	2.49	3	1
1:A:969:PHE:CE1	1:A:997:PHE:CE1	0.40	3.09	10	1
1:A:969:PHE:CD1	1:A:970:ARG:N	0.40	2.89	5	1
1:A:922:LEU:HD13	1:A:922:LEU:C	0.40	2.37	3	1
1:A:969:PHE:O	1:A:973:PHE:CE2	0.40	2.74	10	1

6.3 Torsion angles (i)

6.3.1 Protein backbone (i)

In the following table, the Percentiles column shows the percent Ramachandran outliers of the chain as a percentile score with respect to all PDB entries followed by that with respect to all NMR entries. The Analysed column shows the number of residues for which the backbone conformation was analysed and the total number of residues.

Mol	Chain	Analysed	Favoured	Allowed	Outliers	Percentiles	
1	A	104/106 (98%)	91±2 (87±2%)	12±2 (12±2%)	1±1 (1±1%)	18	66
All	All	1040/1060 (98%)	909 (87%)	120 (12%)	11 (1%)	18	66

PRELIMINARY VALIDATION REPORT

All 7 unique Ramachandran outliers are listed below. They are sorted by the frequency of occurrence in the ensemble.

Mol	Chain	Res	Type	Models (Total)
1	A	957	SER	3
1	A	925	GLN	2
1	A	958	MET	2
1	A	955	ASP	1
1	A	976	CYS	1
1	A	979	PHE	1
1	A	971	LEU	1

6.3.2 Protein sidechains (i)

In the following table, the Percentiles column shows the percent sidechain outliers of the chain as a percentile score with respect to all PDB entries followed by that with respect to all NMR entries. The Analysed column shows the number of residues for which the sidechain conformation was analysed and the total number of residues.

Mol	Chain	Analysed	Rotameric	Outliers	Percentiles	
1	A	100/100 (100%)	91±2 (91±2%)	9±2 (9±2%)	14	61
All	All	1000/1000 (100%)	914 (91%)	86 (9%)	14	61

All 32 unique residues with a non-rotameric sidechain are listed below. They are sorted by the frequency of occurrence in the ensemble.

Mol	Chain	Res	Type	Models (Total)
1	A	930	LEU	8
1	A	997	PHE	7
1	A	932	VAL	6
1	A	969	PHE	6
1	A	956	TYR	5
1	A	943	MET	5
1	A	924	PHE	4
1	A	945	LEU	4
1	A	952	LEU	3
1	A	979	PHE	3
1	A	970	ARG	3
1	A	951	ARG	2
1	A	935	TYR	2
1	A	961	LYS	2
1	A	958	MET	2
1	A	925	GLN	2
1	A	949	LYS	2

Continued on next page...

PRELIMINARY VALIDATION REPORT

Continued from previous page...

Mol	Chain	Res	Type	Models (Total)
1	A	920	MET	2
1	A	972	ILE	2
1	A	981	GLU	2
1	A	978	GLU	2
1	A	950	LYS	2
1	A	955	ASP	1
1	A	985	GLU	1
1	A	931	THR	1
1	A	922	LEU	1
1	A	983	ASP	1
1	A	968	ASP	1
1	A	980	ASN	1
1	A	965	PHE	1
1	A	940	LYS	1
1	A	939	ILE	1

6.3.3 RNA (i)

There are no RNA molecules in this entry.

6.4 Non-standard residues in protein, DNA, RNA chains (i)

There are no non-standard protein/DNA/RNA residues in this entry.

6.5 Carbohydrates (i)

There are no monosaccharides in this entry.

6.6 Ligand geometry (i)

Of 1 ligands modelled in this entry, 1 could not be matched to an existing wwPDB Chemical Component Dictionary definition at this stage - leaving 0 for Mogul analysis.

6.7 Other polymers (i)

There are no such molecules in this entry.

PRELIMINARY VALIDATION REPORT

6.8 Polymer linkage issues (i)

There are no chain breaks in this entry.

PRELIMINARY VALIDATION REPORT

7 Chemical shift validation

The completeness of assignment taking into account all chemical shift lists is 0% for the well-defined parts and 0% for the entire structure.

7.1 Chemical shift list 1

File name: D_1292111532_cs_P1.cif.V11

Chemical shift list name: *assigned_chem_shift_list*

7.1.1 Bookkeeping

The following table shows the results of parsing the chemical shift list and reports the number of nuclei with statistically unusual chemical shifts.

Total number of shifts	62
Number of shifts mapped to atoms	0
Number of unparsed shifts	62
Number of shifts with mapping errors	0
Number of shifts with mapping warnings	0
Number of shift outliers (ShiftChecker)	0

The following errors were found when reading this chemical shift list.

- Entity instance (chain) must be specified. All 62 occurences are reported below.

Shift ID	Chain	Res	Type	Atom	Shift Data		
					Value	Uncertainty	Ambiguity
1	?	22	LEU	HD21	1.076	0.0	2
2	?	22	LEU	HD22	1.076	0.0	2
3	?	22	LEU	HD23	1.076	0.0	2
4	?	1	JO2	H26	0.817	0.003	1
5	?	1	JO2	H27	0.817	0.003	1
6	?	1	JO2	H25	0.817	0.003	1
7	?	1	JO2	H24	1.607	0.007	2
8	?	1	JO2	H23	1.533	0.009	2
9	?	1	JO2	H9	7.338	0.004	1
10	?	1	JO2	H22	3.760	0.003	2
11	?	1	JO2	H4	7.600	0.003	1
12	?	1	JO2	H18	7.011	0.002	1
13	?	1	JO2	H8	6.903	0.003	1
14	?	1	JO2	H19	6.463	0.002	1
15	?	1	JO2	H20	5.851	0.003	1

Continued on next page...

PRELIMINARY VALIDATION REPORT

Continued from previous page...

Shift ID	Chain	Res	Type	Atom	Shift Data		
					Value	Uncertainty	Ambiguity
16	?	1	JO2	H14	3.004	0.003	1
17	?	1	JO2	H13	3.004	0.003	1
18	?	1	JO2	H12	3.004	0.003	1
19	?	1	JO2	H16	3.316	0.002	1
20	?	1	JO2	H17	3.316	0.002	1
21	?	1	JO2	H15	3.316	0.002	1
22	?	1	JO2	H30	4.105	0.004	2
23	?	1	JO2	H29	4.036	0.007	2
24	?	1	JO2	H32	1.939	0.018	2
25	?	1	JO2	H31	1.833	0.003	2
26	?	1	JO2	H33	3.073	0.006	2
27	?	1	JO2	H34	3.120	0.005	2
28	?	1	JO2	H11	7.818	0.003	1
29	?	1	JO2	H7	3.831	0.005	1
30	?	1	JO2	H6	3.831	0.005	1
31	?	1	JO2	H5	3.831	0.005	1
32	?	1	JO2	H3	3.714	0.002	1
33	?	1	JO2	H1	3.714	0.002	1
34	?	1	JO2	H2	3.714	0.002	1
35	?	1	JO2	H21	3.399	0.003	2
36	?	23	ALA	HB2	0.149	0.001	1
37	?	23	ALA	HB3	0.149	0.001	1
38	?	23	ALA	HB1	0.149	0.001	1
39	?	86	VAL	HG11	0.488	0.001	2
40	?	86	VAL	HG12	0.488	0.001	2
41	?	86	VAL	HG13	0.488	0.001	2
42	?	86	VAL	HG21	0.580	0.001	2
43	?	86	VAL	HG22	0.580	0.001	2
44	?	86	VAL	HG23	0.580	0.001	2
45	?	89	ALA	HB2	1.830	?	1
46	?	89	ALA	HB3	1.830	?	1
47	?	89	ALA	HB1	1.830	?	1
48	?	20	MET	HE3	2.167	?	1
49	?	20	MET	HE1	2.167	?	1
50	?	20	MET	HE2	2.167	?	1
51	?	28	VAL	HG11	-0.013	0.0	2
52	?	28	VAL	HG12	-0.013	0.0	2
53	?	28	VAL	HG13	-0.013	0.0	2
54	?	22	LEU	HD11	1.037	0.0	2
55	?	22	LEU	HD12	1.037	0.0	2
56	?	22	LEU	HD13	1.037	0.0	2

Continued on next page...

PRELIMINARY VALIDATION REPORT

Continued from previous page...

Shift ID	Chain	Res	Type	Atom	Shift Data		
					Value	Uncertainty	Ambiguity
57	?	28	VAL	HG21	1.129	0.001	2
58	?	28	VAL	HG22	1.129	0.001	2
59	?	28	VAL	HG23	1.129	0.001	2
60	?	31	THR	HG22	1.206	0.001	1
61	?	31	THR	HG23	1.206	0.001	1
62	?	31	THR	HG21	1.206	0.001	1

7.1.2 Chemical shift referencing (i)

No chemical shift referencing corrections were calculated (not enough data).

7.1.3 Completeness of resonance assignments (i)

The following table shows the completeness of the chemical shift assignments for the well-defined regions of the structure. The overall completeness is 0%, i.e. 0 atoms were assigned a chemical shift out of a possible 1386. 0 out of 17 assigned methyl groups (LEU and VAL) were assigned stereospecifically.

	Total	^{1}H	^{13}C	^{15}N
Backbone	0/514 (0%)	0/204 (0%)	0/212 (0%)	0/98 (0%)
Sidechain	0/745 (0%)	0/441 (0%)	0/274 (0%)	0/30 (0%)
Aromatic	0/127 (0%)	0/67 (0%)	0/58 (0%)	0/2 (0%)
Overall	0/1386 (0%)	0/712 (0%)	0/544 (0%)	0/130 (0%)

The following table shows the completeness of the chemical shift assignments for the full structure. The overall completeness is 0%, i.e. 0 atoms were assigned a chemical shift out of a possible 1386. 0 out of 17 assigned methyl groups (LEU and VAL) were assigned stereospecifically.

	Total	^{1}H	^{13}C	^{15}N
Backbone	0/514 (0%)	0/204 (0%)	0/212 (0%)	0/98 (0%)
Sidechain	0/745 (0%)	0/441 (0%)	0/274 (0%)	0/30 (0%)
Aromatic	0/127 (0%)	0/67 (0%)	0/58 (0%)	0/2 (0%)
Overall	0/1386 (0%)	0/712 (0%)	0/544 (0%)	0/130 (0%)

7.1.4 Statistically unusual chemical shifts (i)

There are no statistically unusual chemical shifts.

PRELIMINARY VALIDATION REPORT

7.1.5 Random Coil Index (RCI) plots (i)

No *random coil index* (RCI) plot could be generated from the current chemical shift list (assigned_chem_shift_list). RCI is only applicable to proteins.

PRELIMINARY VALIDATION REPORT
